# Mapping of Motor Function with Neuronavigated Transcranial Magnetic Stimulation: A Review on Clinical Application in Brain Tumors and Methods for Ensuring Feasible Accuracy

**DOI:** 10.3390/brainsci11070897

**Published:** 2021-07-07

**Authors:** Nico Sollmann, Sandro M. Krieg, Laura Säisänen, Petro Julkunen

**Affiliations:** 1 Department of Diagnostic and Interventional Radiology, University Hospital Ulm, Albert-Einstein-Allee 23, 89081 Ulm, Germany; 2 Department of Diagnostic and Interventional Neuroradiology, School of Medicine, Klinikum rechts der Isar, Technical University of Munich, Ismaninger Str. 22, 81675 Munich, Germany; 3 TUM-Neuroimaging Center, Klinikum rechts der Isar, Technical University of Munich, 81675 Munich, Germany; sandro.krieg@tum.de; 4 Department of Radiology and Biomedical Imaging, University of California San Francisco, 185 Berry Street, San Francisco, CA 94143, USA; 5 Department of Neurosurgery, School of Medicine, Klinikum rechts der Isar, Technical University of Munich, Ismaninger Str. 22, 81675 Munich, Germany; 6 Department of Clinical Neurophysiology, Kuopio University Hospital, 70029 Kuopio, Finland; laura.saisanen@kuh.fi (L.S.); Petro.Julkunen@kuh.fi (P.J.); 7 Department of Applied Physics, University of Eastern Finland, 70211 Kuopio, Finland

**Keywords:** brain stimulation, brain tumor, electric field, eloquent cortex, functional mapping, motor mapping, motor threshold, navigated transcranial magnetic stimulation, neuronavigation, presurgical evaluation

## Abstract

Navigated transcranial magnetic stimulation (nTMS) has developed into a reliable non-invasive clinical and scientific tool over the past decade. Specifically, it has undergone several validating clinical trials that demonstrated high agreement with intraoperative direct electrical stimulation (DES), which paved the way for increasing application for the purpose of motor mapping in patients harboring motor-eloquent intracranial neoplasms. Based on this clinical use case of the technique, in this article we review the evidence for the feasibility of motor mapping and derived models (risk stratification and prediction, nTMS-based fiber tracking, improvement of clinical outcome, and assessment of functional plasticity), and provide collected sets of evidence for the applicability of quantitative mapping with nTMS. In addition, we provide evidence-based demonstrations on factors that ensure methodological feasibility and accuracy of the motor mapping procedure. We demonstrate that selection of the stimulation intensity (SI) for nTMS and spatial density of stimuli are crucial factors for applying motor mapping accurately, while also demonstrating the effect on the motor maps. We conclude that while the application of nTMS motor mapping has been impressively spread over the past decade, there are still variations in the applied protocols and parameters, which could be optimized for the purpose of reliable quantitative mapping.

## 1. Introduction

**Figure 1:**
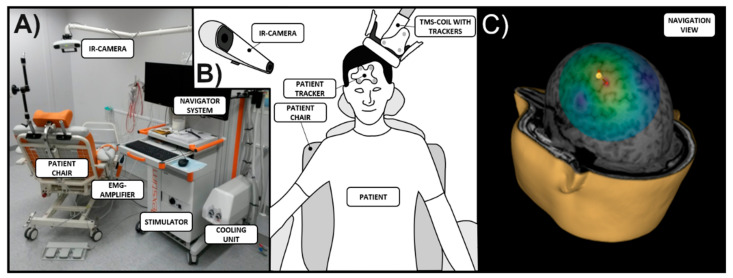
Setup for nTMS mapping. (**A**) Hardware of the nTMS system, including an infrared camera, EMG amplifier, neuronavigation monitor, and the stimulator with a cooling unit as the central components. (**B**) Patient positioning during mapping procedures, requiring initial co-registration of the patient’s head with cranial MRI (using a head tracker attached to the patient’s forehead) to be able to track the stimulating coil (equipped with infrared trackers) in relation to individual brain anatomy. (**C**) Navigation view during the mapping procedure, showing the stimulating coil (*yellow marker*) with its orientation (*red arrow*) and modelled EF distribution.

Navigated transcranial magnetic stimulation (nTMS) combines the use of neuronavigation with TMS to target neurostimulation inductively to the brain cortex, utilizing views of the brain anatomy with sub-centimeter precision and enabling tracking of the coil during stimulation (e.g., using an infrared (IR) tracking system combined with the stimulator; Figure 1) [1]. For over a decade, nTMS has been used in diagnostic setups (e.g., to perform non-invasive clinical mapping of motor or other brain functions) or for therapeutic purpose (e.g., to treat major depression or other psychiatric diseases as well as chronic pain). Preceding nTMS as we know it today, early approaches conducted mapping based on anatomical landmarks placed over the scalp or used frameless stereotactic systems to localize and track stimulations [2,3,4]. However, such approaches seemed limited for clinical use where high precision and reliability is warranted in relation to individual brain anatomy that can be both spatially and functionally deranged due to pathology. Technological advancements have enabled the estimation of the spatial extent and geometry of the electric field (EF) induced by stimulation [1,5,6,7]. Specifically, the development of nTMS using EF-based neuronavigation acknowledges that the EF induced by stimulation strongly depends on several factors such as skull thickness and coil tilting, amongst others. In this context, accurate and efficient modelling of the EF is essential to pinpoint the impact of stimulation and to understand its effects [1,5,6,7]. Since nTMS using such EF-based neuronavigation is currently considered the most accurate method for targeting stimulation, this article focusses on this technique.

**Figure 2:**
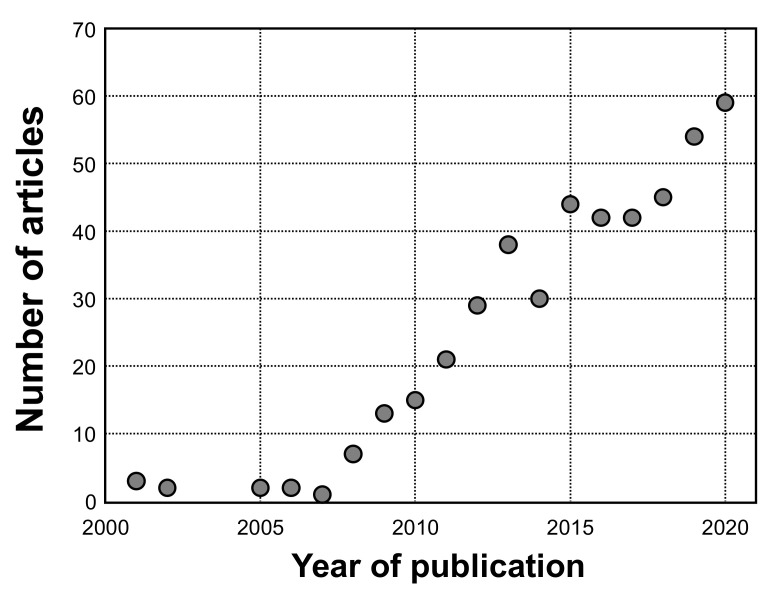
Number of articles on nTMS published annually between 2002 and 2020 as indexed in PubMed (on the 21st of May in 2021). The search query in PubMed was defined as follows: (“navigated transcranial magnetic stimulation” OR “navigated TMS” OR “neuronavigated TMS” OR “neuronavigated transcranial magnetic stimulation”).

Overall, interest towards combining neuronavigation with TMS has experienced more and more interest in the research community as indicated by the increasing trend in published studies (Figure 2). From a clinical perspective, nTMS has been gaining importance especially as a tool for preoperative mapping used for planning and intraoperative resection guidance in patients harboring functionally eloquent brain neoplasms [1,8,9,10,11]. As of today, mapping of motor function has become the mainstay of nTMS, making so-called nTMS motor mapping in patients with motor-eloquent lesions an ideal use case for the technique. In modern neuro-oncological surgery, achieving an optimal balance between the extent of resection (EOR) of a brain tumor and the individual functional status of the patient is the major principle for surgical resection [12]. Prognostically, a safely performed maximized tumor resection is of utmost importance as incomplete resection is correlated to lower survival rates and quality of life for patients with malignant glioma as the prominent entity of intra-axial brain tumors [13,14,15,16,17,18]. To maximize the EOR while keeping surgery-related decline of function (e.g., paresis in the context of the motor system) to the lowest level achievable, intraoperative direct electrical stimulation (DES) is performed as the gold-standard method for assessing subcortical and cortical functional representations [19,20,21,22,23]. Outside of the operating theater, nTMS motor mapping and later-developed nTMS-based tractography can be performed for this purpose. As the major field of current application, the first part of this article focusses on the clinical utility of nTMS-based motor mapping and derived tractography in patients with motor-eloquent brain neoplasms.

**Figure 3:**
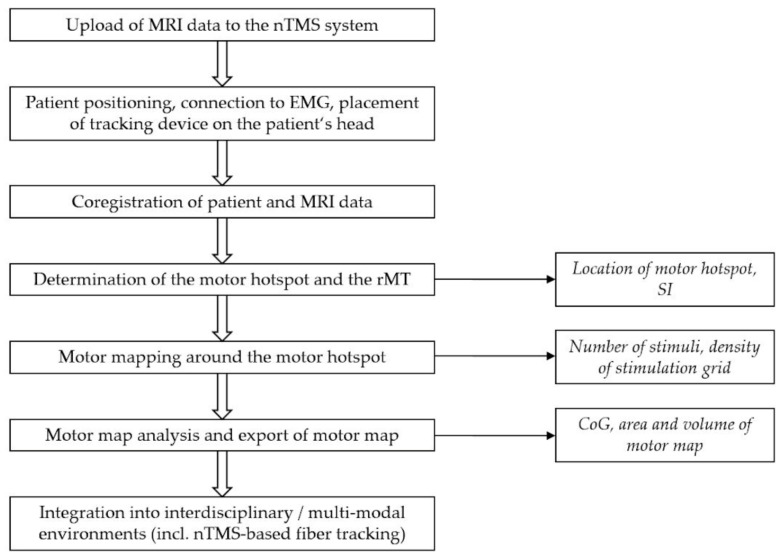
Overview of the main steps during the nTMS mapping procedure, starting with upload of the MRI data to the nTMS system and resulting in the generation of the individual motor map that can be further used for nTMS-based fiber tracking and other interdisciplinary or multi-modal applications. In addition, the main quantitative parameters that can be extracted from the single steps are shown (including the location of the motor hotspot, SI, number of stimuli, density of the stimulation grid, CoG, area, and volume of the motor map).

From a methodological perspective, the nTMS parameters selected for motor mapping, such as the stimulation intensity (SI), number of applied stimuli, and the density of the applied stimulation grid, affect the outcome and accuracy of the motor mapping (Figure 3) [24,25,26,27]. Specifically, optimal selection becomes especially important when quantitative parameters are drawn from the motor maps, e.g., to characterize the location of motor function or to define its extent on the cortex. Quantitative mapping refers to those quantitative parameters derived from nTMS motor mapping, based on the recorded responses and location information related to the responses. The quantitative parameters in nTMS motor mapping include the locations of the motor hotspot and center of gravity (CoG), and area and volume of the motor map, which are all based on recorded amplitudes of motor-evoked potentials (MEPs) elicited by stimulation (Figure 3) [24,25,26,27]. The motor hotspot location is often considered as the location of the maximum MEP amplitude response [28,29]. It not only characterizes the major location of the motor representation, but is also used as the location where the resting motor threshold (rMT) is determined and around which the motor map extends. The CoG is defined as the amplitude-weighted location representing the location in the motor representation area where the center of motor activation lies [28,30,31]. The volume of the motor map is usually calculated by summing the MEP amplitudes within a motor map, e.g., within a stimulus grid [30,31,32,33]. The area of the motor map on the other hand binarizes the MEP responses to positive and negative responses and represents the area covered by positive responses (i.e., spots where stimulation elicited an MEP with a certain amplitude) [27,34,35]. Importantly, wrong selection or wrong interpretation of these quantitative parameters affects the confidence in the motor map and, in the worst case, could lead to imprecise motor maps that may have harmful consequences to the patient when used for preoperative planning and intraoperative resection guidance in neuro-oncological surgery. The technical solutions and refinements realized in the nTMS systems by the manufacturers are also important for the overall performance of motor mapping, albeit representative comparisons between different manufacturer-specific approaches have not yet been made in the literature. Given the crucial role quantitative parameters play for nTMS motor mapping, the second part of this article demonstrates the significance of certain parameters used in motor mapping to apply quantitative measures on the resulting individual motor maps. Finally, we speculate on the future of nTMS motor mapping by the potential in advancing from current trends towards purely quantitative motor mapping, which would have direct clinical impact. Relevant studies were identified by PubMed search (http://www.ncbi.nlm.nih.gov/pubmed; accessed on 21 May 2021).

## 2. Clinical Application of nTMS for Mapping Motor Function

### 2.1. Feasibility, Reliability, and Comparison with Other Methods

The gold standard for functional brain mapping is defined by DES. Thus, any other technique for mapping purposes needs to be validated against it. First case reports in patients with intracranial metastasis (MET), meningioma (MEN), and low-grade glioma (LGG) have already indicated good agreement between preoperative motor mapping by nTMS and DES [32,33,34]. Specifically, in an early series of 20 patients with different entities of intracranial neoplasms, the motor hotspot was located on the same gyrus for nTMS and DES mappings in all enrolled cases, with distances of motor hotspots between techniques amounting to values <1 cm for specific cortical muscle representations [35]. In this regard, Table 1 provides an overview of studies on reliability of nTMS motor mapping and comparison with other methods, mainly including DES.

The initial impression of high concordance between motor maps provided by preoperatively acquired nTMS motor mapping and those derived from DES was confirmed by subsequent studies that aimed to enroll larger patient cohorts with different entities of brain lesions and performed comparisons with a third method. Specifically, comparative analyses were achieved for nTMS and task-based functional magnetic resonance imaging (fMRI) versus DES mappings since task-based fMRI appeared as a widely used alternative to nTMS, with all major studies consistently revealing closer distances for localizations of cortical motor function between preoperative nTMS and DES compared with task-based fMRI activation maps [36,37,38,39,40,41]. Exemplarily, in a series of 26 patients, distances between motor hotspots of nTMS and DES amounted to 4.4 ± 3.4 mm on average [38]. However, when comparing distances between activation in task-based fMRI and nTMS motor hotspots, larger distances of 9.8 ± 8.5 mm for upper extremity (uE) and 14.7 ± 12.4 mm for lower extremity (lE) muscle representations were observed [38]. More recently, considering motor representations of the uE, lE, and tongue in 36 patients with intracranial tumors, there were significantly smaller Euclidean distances as well as a better spatial overlap between DES and nTMS compared with task-based fMRI [41]. In this regard, one study suggested that nTMS motor mapping is as accurate in recurrent gliomas as it seems to be prior to the initial surgery, opening up the possibility to reuse nTMS motor mapping in the individual course of disease [39]. Similar to results for nTMS motor mapping versus fMRI, in an investigation comparing the accuracy of nTMS with magnetoencephalography (MEG) and DES mapping, distances between motor hotspots of nTMS and DES mappings were significantly smaller than those between the activation in MEG and nTMS motor hotspots [42].

The implementation of nTMS motor mapping confirmed the expected anatomy in 22%, added awareness of high-risk functional areas in 27%, modified the approach in 16%, changed the planned EOR in 8%, and changed the surgical indication in 3% of patients among a cohort of 73 patients with different entities of brain neoplasms [43]. Similarly, another study concluded that nTMS motor mapping enabled an exact localization of the motor cortex in 88.2%, provided the neurosurgeon with new unexpected information about functional anatomy in 70.6%, and facilitated a modification of the surgical approach to spare the motor cortex from damage in 29.4% of patients [44]. Further, the availability of nTMS data improved the neurosurgeons’ confidence in identifying the motor strip or central region [38,40]. Importantly, valuable motor mapping data from nTMS may be generated irrespective of the distinct experience level of the operator (after a certain interval of training), with average intra-examiner distances between CoGs for an expert investigator amounting to 4.4 mm and 4.9 mm for the expert versus novice investigator, with comparable values in the investigated healthy subjects as well as in patients with brain tumors [45].

Regarding potential variability of MEP latencies or the SI applied during nTMS motor mapping as a function of the rMT, it has been revealed in large cohorts of patients with motor-eloquent tumors that sex and antiepileptic drug (AED) intake, amongst other factors, may considerably contribute to MEP latency variability as well as variability of the determined rMT [46,47]. Interestingly, also the tumor grading according to the World Health Organization (WHO) scheme may be held accountable for a certain degree of variability introduced to nTMS motor mappings because MEP latencies of lE muscles increased with the WHO grading, and correlations between the increase in WHO grading and a decreased rMT were observed for lE muscles [48]. From a more methodological perspective, efforts have been made to expand accurate nTMS-based motor mapping beyond the limits of the supposed primary motor cortex, and to increase robustness by introducing altered threshold values for MEP amplitudes [49,50]. Future research will determine the distinct relevance of such influencing factors for motor mapping by nTMS in patients with brain tumors, in whom such potential confounders of motor mapping results are still largely neglected in the clinical routine workflow.

### 2.2. Fiber Tractography

**Figure 4:**
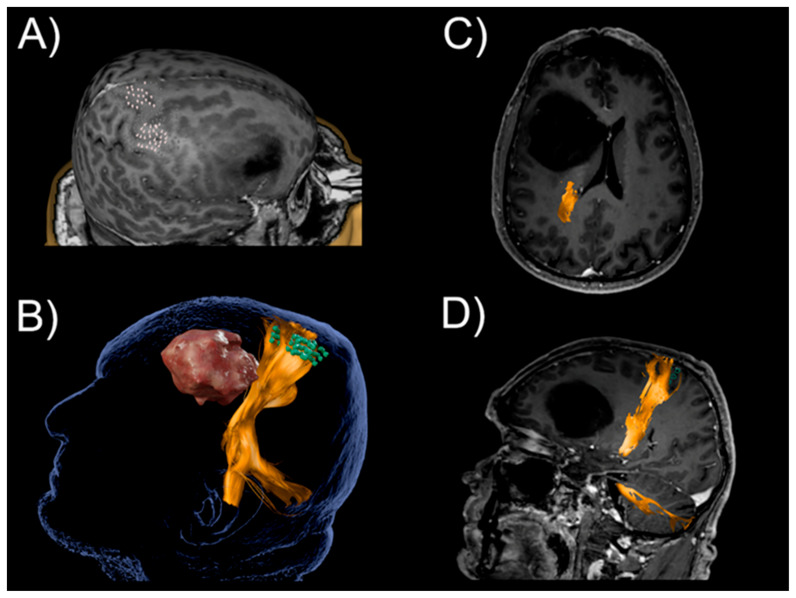
Exemplary patient case (right-hemispheric glioma in a 56-year-old male patient) for illustration of CST reconstruction using tractography based on motor maps derived from motor mapping with nTMS. (**A**) Motor map with binarization into motor-positive (*white*) and motor-negative (*grey*) stimulation points. (**B**) Tractography of the CST (*orange*) based on a ROI constituted of motor-positive nTMS points (*green*). (**C**) Fusion of T1-weighted imaging and tractography results (axial plane). (**D**) Fusion of T1-weighted imaging and tractography results (sagittal plane).

The motor-positive nTMS points can be used for seeding to obtain regions of interest (ROIs) for subsequent delineation of the corticospinal tract (CST) in the context of fiber tracking. The combination of nTMS motor maps at the cortical surface with diffusion MRI (dMRI) and tractography, i.e., the delineation of the primary motor cortex and its subcortical connections to the peripheral nervous system with visualization of the course of the CST, can provide a more complete picture than one of the techniques alone (Figure 4). In this regard, Table 2 outlines studies using data from nTMS motor mapping for tractography of the CST in patients with different kinds of brain neoplasms.

In a first tractography study using nTMS data, 30 patients harboring motor-eloquent brain tumors underwent presurgical nTMS motor mapping followed by nTMS-based tractography, using the nTMS motor-positive spots and the ipsilateral cerebral peduncle as ROIs [51]. Compared with the conventionally used approach with manual delineation of the suspected primary motor cortex and the ipsilateral cerebral peduncle as ROIs, the novel setup led to a lower number of fibers displayed, a reduced volume of the CST, and, most importantly, lower fractions of aberrant tracts most likely not belonging to the CST [51]. The approach of nTMS-based tractography of the CST was subsequently refined by investigating individually adapted adjustments for the fractional anisotropy (FA) that had to be defined for the deterministic tractography algorithm: the FA was increased stepwise until no fibers were displayed, followed by reducing the FA value by 0.01, thus delineating only a thin fiber course; the obtained FA value was defined as 100% FA threshold (FAT), and nTMS-based tractography was then carried out with 50% and 75% FAT with motor-positive nTMS points and the manually delineated internal capsule or brainstem as ROIs [52]. This method influenced the surgical strategy in 46% of patients, in contrast to conventional tractography without nTMS data for ROI generation where an impact was only observed for 22% of patients [52].

Furthermore, a study among 20 patients with different entities of brain neoplasms achieved detailed somatotopic CST reconstruction derived from nTMS motor maps combined with dMRI for uE, lE, and face muscle representations, with a decreased number of fibers and a greater overlap between the motor cortex and the cortical end-region of the CST when compared with conventional tractography with only anatomical seeding [53]. Of note, the obtained CST course as well as the somatotopic organization were confirmed by DES mapping [53]. In another study on somatotopic reconstruction of the CST considering parts subserving uE, lE, and face muscles, a higher fraction of plausible fibers was observed for seeding at the anterior inferior pontine level when compared with seeding at the internal capsule, combined with nTMS-based seeding at the cortical level [54]. When setting somatotopic nTMS-based tractography in contrast to fMRI-based seeding, a higher plausibility was observed for the nTMS-based approach, and fMRI-originated tracts presented with a more posterior course relative to the nTMS-based reconstruction of tracts [55]. Recently, in a comprehensive study systematically comparing different setups for tractography (deterministic and probabilistic algorithms with variable ROI definitions) and correlating tractography with DES mapping and fMRI findings in 11 adult patients, highest accuracy of tractography was achieved when using seeding with a manually generated mask enclosing the precentral gyrus, but none of the applied setups showed clear superiority and nTMS- or fMRI-based tractography differed only slightly [56]. Yet, probabilistic tracking resulted in an optimized correlation with DES mapping when compared with the more commonly used deterministic tractography algorithm [56]. Upcoming work is needed to further investigate optimal settings and algorithms in representative cohort sizes to achieve results of CST tractography as close as possible to DES mapping results in order to assure accuracy and reliability of nTMS-based tractography.

### 2.3. Improvement of Clinical Outcome

When added to the armamentarium of the preoperative workup of patients harboring brain neoplasms, the question arises whether nTMS motor mapping and derived nTMS-based tractography may be capable of improving the clinical outcome, as measured by an ideally increased EOR combined with lowered rates of functional perioperative decline. In this regard, Table 3 gives an overview of studies focused on clinical outcome.

An initial study compared 11 patients who underwent preoperative nTMS to 11 patients without nTMS motor mapping, revealing that preoperative nTMS motor mapping changed the treatment plan towards early and more extensive resection in 6 out of 11 patients [57]. Furthermore, one of four patients of the nTMS group with preoperative motor deficits improved by one year, whereas increased deficits were observed in three of the eight patients of the historical group not having surgery [57]. In two retrospective studies in considerably large cohorts of patients with different types of brain tumors, the utility of nTMS motor mapping and nTMS-based tractography of the CST may have facilitated a more extensive EOR, combined with tendencies towards better motor function after surgery [58,59]. Specifically, nTMS disproved suspected involvement of the primary motor cortex in 25.1% of the 250 enrolled patients, and it enabled expanding surgical indication in 14.8%, thus facilitating planning of more extensive resections in 35.2% of patients [58]. Furthermore, the distinct add-on value of nTMS-based tractography of the CST has been evaluated in a study including 70 adult patients with different brain tumor entities, revealing that patients having nTMS-based tractography available are characterized by an improved risk-benefit profile, showed an increased EOR, and demonstrated reduced rates of worsening in motor function in cases of already preexisting preoperative motor deficits [60].

In a follow-up study investigating the role of nTMS motor mapping and nTMS-based tractography in 70 patients presenting with high-grade glioma (HGG), residual tumor tissue and unexpected tumor residuals were less frequent in the nTMS group compared with historical control patients, with patients of the nTMS group being more frequently eligible for postoperative radiotherapy and showing prolonged 3-, 6-, and 9-month survival rates [61]. A significantly higher EOR was subsequently confirmed by another study for patients with a diagnosis of glioblastoma multiforme (GBM), in which patients of the nTMS group showed a gross total resection (GTR) rate of 61% versus 45% for the non-nTMS group [62]. Analogously, in studies focusing on patients with intracranial MET, patients of the nTMS group showed a lower rate of residuals combined with comparatively low rates of perioperative decline of motor function [63,64]. Specifically, in a retrospective comparative study pooling patients with intracranial MET from three different neurosurgical centers, surgery-related paresis was clearly less frequent in patients of the nTMS group [64]. In 47 patients with MEN located in the rolandic area, nTMS motor mapping and tractography facilitated a modification of the surgical strategy in 42.5% of cases, and a new permanent motor deficit (i.e., deficit that did not resolve to the preoperative status within the follow-up interval) occurred in 8.5% of cases, which is at the lower edge of the range for motor deficits known from the literature of the pre-nTMS era [65]. Furthermore, the combination of sodium fluorescein-guided resection (FGR) with preoperative nTMS motor mapping and tractography has been explored recently, revealing a higher GTR rate for patients operated on using nTMS and FGR as well as lower rates of new surgery-related permanent motor deficits when compared with controls [66,67].

One study in 43 patients with LGG and HGG showed that 72% of patients had motor-positive nTMS points in areas frontal of the rolandic area and, thus, outside of the expected spatial dimensions of the primary motor cortex [68]. Interestingly, 10 of the 13 patients who underwent resection of motor-positive nTMS points presented with postoperative paresis (8 patients with a new permanent surgery-related paresis), suggesting that even motor-positive nTMS points within the superior or middle frontal gyrus should be considered carefully for resection planning and guidance to avoid perioperative functional decline [68]. Hence, nTMS motor mapping and derived tractography may help to understand individual functional anatomy, allowing for optimized resection that provides a high EOR and low rates of surgically induced motor function decline.

### 2.4. Risk Stratification and Prediction

Besides the role for preoperative planning and intraoperative resection guidance, nTMS motor mapping and tractography could also be efficiently used for risk stratification and prediction of the motor status in patients with brain neoplasms. This has already been acknowledged by a growing body of studies, which are summarized in Table 4.

An early study characterized the neurophysiological status as derived from nTMS motor mapping in 100 patients, and already suggested that interhemispheric differences for MEP latencies may be considered as potential warning signs for the motor system at risk as comparatively similar latencies are commonly observed between the two hemispheres [69]. On a similar note, a high interhemispheric rMT ratio (i.e., the ratio between the two hemispheres regarding the rMT, which is commonly higher in a tumor-affected hemisphere) could suggest immanent deterioration of the functional motor status [69]. Furthermore, two studies in patients with various tumor entities investigated the role of nTMS-based tractography of the CST for risk stratification, evaluating the cut-off value for the lesion-to-CST distance that amounted to 8 mm and 12 mm to avoid new surgery-related permanent motor deficits, respectively [70,71]. Hence, patients that showed a lesion-to-CST distance above this cut-off value based on preoperative nTMS-based tractography were unlikely to suffer from surgery-related postoperative permanent paresis [70,71]. Moreover, statistically significant negative correlations were observed between the rMT value and lesion-to-CST distances in patients with a new surgery-related paresis, emphasizing the interplay between the SI used during motor mapping and results of nTMS-based tractography [71]. Correspondingly, motor function did not improve in cases with the rMT being significantly higher in the tumor-affected hemisphere than in the contralateral hemisphere, as expressed by an interhemispheric rMT ratio of >110% [70]. In a study investigating patients harboring HGG, lower FA values within the tumor-affected CST and higher average apparent diffusion coefficient (ADC) values were significantly correlated to worsened postoperative motor function, thus further exploring the contribution of dMRI-derived metrics to risk modelling [72].

In an innovative approach investigating postoperative nTMS motor mapping—instead of standardly used presurgical mapping—compared with intraoperative neuromonitoring (IONM) for predicting recovery of motor function, it was revealed that IONM and postoperative nTMS motor mapping were equally predictive for long-term motor recovery [73]. Specifically, when postoperative motor mapping was able to elicit MEPs, motor strength recovered to a score of at least 4/5 on the British Medical Research Council (BMRC) scale within one month after surgery, whereas when postoperative nTMS motor mapping did not elicit MEPs, the patient did not recover [73]. Furthermore, when implementing presurgical nTMS motor mapping and tractography in multi-modal neuroimaging with multi-sequence MRI and dedicated positron emission tomography (PET) protocols, it has been demonstrated that PET may be superior to contrast-enhanced T1-weighted MRI for proposing a motor deficit prior to surgery, and that the highest association with clinical impairment was revealed for the T2-weighted lesion overlap with functional brain tissue (i.e., the spatial overlap between the lesion volume on T2-weighted images of MRI and the functional primary motor cortex and/or CST volumes as derived from nTMS motor mapping and nTMS-based tractography) [74]. Future research may further explore the role of nTMS in multi-modal environments, given that data from various methods are frequently available for clinical needs prior to surgery. Opportunistic use of data from adjunct modalities (e.g., PET) as well as performance of dedicated longitudinal motor mapping (e.g., during the immediate postoperative course and during long-term follow-up examinations) could pave the way for a more efficient use of nTMS motor mapping and related tractography.

### 2.5. Plasticity and Reallocation of Motor Function

Repeated application of nTMS motor mapping and tractography has potential to provide insights into brain plasticity that is likely to occur to a certain degree due to the presence and growth of a brain tumor. Few studies have already tried to investigate the role of nTMS motor mapping in this regard, and these studies are outlined in Table 5.

The non-invasive character of nTMS makes possible the acquisition of data from multiple time points, ideally spanning from the preoperative to the postoperative and follow-up interval. Correspondingly, an early explorative study in five patients and five controls used preoperative motor mapping by nTMS as well as mapping during follow-up examinations, revealing a shift of CoGs over a mean interval of 18 months of 6.8 ± 3.4 mm and a shift of motor hotspots of 8.7 ± 5.1 mm for the dominant hemispheres [75]. In a case report on a patient with a LGG that was situated within the frontal lobe and affected the suspected primary motor cortex, motor representation shifted from the precentral to the postcentral gyrus over an interval of 18 months according to serial nTMS motor mappings, which was confirmed by DES mapping during re-resection [76].

In general, a connection between the distinct location of the motor map as enclosed by nTMS as well as its extent and tumor location has been demonstrated in the sense of tumor location-dependent changes in the distribution of polysynaptic MEP latencies and spread of motor maps, especially along the anterior-posterior direction [77]. In the further course, it was revealed that in a majority of patients with mixed tumor entities, MEP counts, when elicited by nTMS to the precentral gyrus, were higher than average, potentially reflecting robust and less variable motor representations within the primary motor cortex [78]. Additionally, patients with tumors affecting the postcentral gyrus and other parietal areas primarily showed high MEP counts when stimulation by nTMS was delivered to the postcentral gyrus [78]. Hence, functional reorganization patterns seem to be reflected by a reorganization within anatomical constraints, such as of the postcentral gyrus [78]. Using again serial nTMS motor mappings from presurgical and follow-up sessions, the initial observation of CoG or motor hotspot shifts have been confirmed in further series including 22 and 20 patients with different tumor entities, respectively [79,80]. Additionally, motor representations appeared to shift more clearly toward the tumor mass if the lesion was anterior to the rolandic region than if it was located posterior to the rolandic region, and a preferential regrowth pattern of tumor recurrence towards the primary motor cortex and/or CST as defined by nTMS-based motor mapping and tractography has been suggested by exploratory approaches [80,81].

### 2.6. Integration into the Clinical Environment

For broad application of nTMS motor mapping and derived nTMS-based tractography in neuro-surgical oncology, seamless integration into existing hospital infrastructure and processes is key for acceptance and optimal use of generated data. In this regard, a structured workflow has already been proposed [82]. It starts with admission of the patient and when the indication for mapping is made and includes, amongst other steps, transfer of nTMS data to a hospital-intern picture archiving and communication system (PACS) as well as reporting within dedicated masks for the hospital-intern electronic patient charts [82].

An example of inter-disciplinary integration into different systems requiring nTMS data transfer is represented by the versatile use of the motor maps for planning and treatment purposes in radiosurgery and radiotherapy [83,84,85,86,87]. The first published approach achieved easy and reliable integration of nTMS, fMRI, and tractography data for radiosurgery treatment planning, which led to an average radiation dose reduction of 17% to functional brain areas in a cohort with mixed entities of pathologies [83]. Another study approved flawless integration of specifically nTMS data for radiosurgery, which influenced the radiosurgical planning procedure by improving risk-benefit balancing in all cases, achieved dose plan modifications in 81.9%, facilitated treatment indication in 63.7%, and reduced radiation doses in 72.7% of cases [86]. Compared with radiosurgery plans without nTMS data, treatment plans with integration of nTMS data demonstrated a significant decrease in dose to eloquent cortex volume, which was achievable without a reduction of the dose applied to intracranial MET [87]. Moreover, integration of nTMS motor maps with radiotherapy planning software for hypofractioned stereotactic treatment regimens for patients diagnosed with intracranial MET has been proposed, and by constraining the dose applied to the nTMS motor maps outside the planning target volume (PTV) to 15 Gy, the mean dose was significantly reduced from 23.0 Gy to 18.9 Gy, while the mean dose of the PTV increased [85]. Analogously, in patients with HGG, mean dose to the nTMS motor maps was significantly reduced by 14.3% when constraining the dose to nTMS motor areas, while the dose to the PTV was not compromised [84].

Furthermore, integrating nTMS motor mapping in clinical workflows has provided initial evidence for the usefulness of the method for planning of a stereotactic tumor biopsy, performing endoscopic cystoventriculostomy, or facilitating a transparietal approach to the trigone of the lateral ventricle in patients with brain neoplasms [88,89,90]. In a special environment such as the intensive care unit with critically ill patients, an approach for safe and reliable use of nTMS motor mapping has been described recently, yet preliminarily in patients suffering from other diseases than brain tumors (e.g., central cord syndrome after trauma, ischemic or hemorrhagic stroke) [91]. In particular, the use of computed tomography (CT) instead of MRI data may help to establish nTMS motor mapping also in special environments with patients who may only be eligible to undergo CT due to specific infrastructural constraints (e.g., non-availability of timely imaging by MRI) or medical conditions (e.g., specific implanted devices as contraindications for MRI) [91,92]. While this underlines the broad applicability of nTMS motor mapping, which requires little patient interaction while creating valuable data on the motor system in a non-invasive way, high accuracy has to be ensured and other imaging sources than MRI have to be regarded as second-line alternatives in selected cases.

## 3. Methodological Considerations on Application of nTMS Motor Mapping

### 3.1. Current Practices and Protocols

Previous large-scale studies have converged to feasible routines for nTMS motor mapping in clinical practice [93]. As an example, the usual SI is normalized to the rMT, and 110% rMT has become the standard, though also 105% rMT is often used. Alternative methods, such as using Mills–Nithi upper threshold (UT), exist as well [93].

There is large variance in the motor mapping protocols since the studies intend to answer specific research questions. On a similar note, the terminology used is not always clear and uniform. New methods and analysis tools have emerged. In addition to formerly used metrics, new and parallel map measures (e.g., area and volume in defining the extent of motor maps) are increasingly used and reported, which brings variance to the studies and makes them more difficult to compare with each other. For instance, motor map topography based on counting the number of discrete peaks, which in turn was based on MEP amplitudes, was introduced in 2017 [94]. Another measure that seems to be interesting and reliable and may also have clinical relevance is the overlap of the motor representation of muscles, though its potential meaning is not yet fully understood [95,96,97].

When it comes to quantitative mapping, a lot of research on quantitative parameters derived from nTMS motor mapping is ongoing. Despite many motor mapping results in patients (such as greater or smaller area of motor maps, closeness of CoGs, or location and shift of CoGs) have already been published, there is clear need of comparison data on healthy volunteers as a basis against which to evaluate cortical reorganization in clinical populations [98,99]. In addition, the normativity of hemispheric side-to-side differences needs to be ensured before comparison between potentially affected and non-affected hemispheres takes place. Fine-scale topography seems to be complex and variable between subjects. To understand it better, multi-modal approaches would be important to better track and understand nTMS-induced effects across the brain [100,101]. Furthermore, instead of mapping single muscles, the importance of groups of muscles, their synergy, and the role of movements and their relation to posture and biomechanics have been pointed out [102].

It should not be forgotten that the steps during initial mapping to locate a motor hotspot, sometimes called coarse mapping or technical mapping, are an important part of the examination to define the precise and correct hotspot. Regarding motor areas of lE muscle representations, a double-cone coil is recommended to reach deeper [103]. Different coil types hinder direct comparison between studies. The coil orientation may also have impact, which partly depends on the area of interest (uE, lE, or face muscle representations) [104,105,106]. The need for preactivation of muscles is a special issue that needs to be taken into account, particularly for lE and face muscle representations [106]. Another important issue is that when mapping the extent of several muscle representations within a limb, the rMT is usually only determined for one specific small hand muscle (abductor pollicis brevis (APB) or first dorsal interosseous (FDI) muscle), and this is used as a reference for motor mapping of representations of other muscles as well. This could perhaps be tackled by targeted post-hoc analysis [95]. For the lE, there is much more variance when choosing the muscle of primary interest, which should preferably also have the lowest rMT. The clinical importance is, however, mostly unknown.

In the analysis of motor mapping data, large variability in MEP amplitudes is a challenge. Another challenge is that the amplitudes are often small. The usual response criterion for an accepted MEP in rest is often 50 µV [26], but lower amplitude criteria have been successfully applied [107]. Some of the measures need to be normalized to the maximum recorded MEP amplitude. Mapping-related biomarkers of sensorimotor plasticity could be used in the study of pathophysiology of different diseases and an important application is rehabilitation. Based on these reflections on the current status and routine procedures for nTMS motor mapping, in the following we outline the most important quantitative parameters and methods for ensuring feasible accuracy of nTMS motor mapping.

### 3.2. Selecting Stimulation Intensity

The proper SI used in quantitative mapping is conventionally and fundamentally dependent on the rMT, which can be determined in several ways [47,108,109,110,111,112]. These days, the most convenient and most widespread method is adaptive threshold hunting (ATH), which estimates the threshold SI in an iterative fashion with excellent confidence [109,110,113,114,115].

Selection of the SI used in motor mapping overall is a crucial part of the experimental design, as the SI defines the amplitude and spread of the responses, and the spatial accuracy of individual stimuli [25]. In general, the map size increases with the SI [24,25]. A common practice is to use a SI that is related to the individual rMT by percentage increase, i.e., 110% of the rMT. A workshop report including recommendations for nTMS motor mapping in patients harboring brain tumors suggest the use of 105% rMT when mapping the uE muscle representations and 110% (with additional 20 V/m) to map the representations of lE muscle representations [93]. While these are practical solutions easily applied for clinical practice, they are likely to cause protocol-induced variation to the results. The individual input–output characteristics vary with factors such as age [116,117]. Furthermore, they are then also affecting the outcome of the mapping, as the used supra-threshold SI is dependent on those characteristics [24,25,107,118,119].

**Figure 5:**
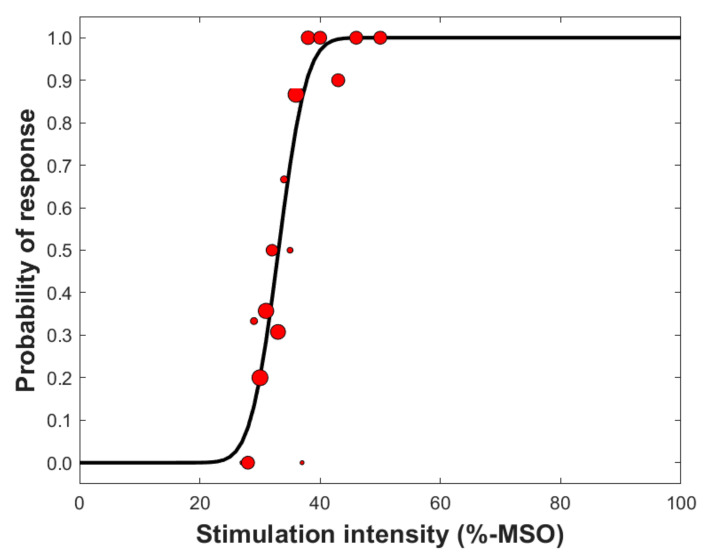
Example of the cumulative distribution function (*black line*) fitted to the experimental data of one subject. The *red dots* indicate the probability of response based on repeated MEP trials. The number of repetitions at each SI (given as %-MSO) is reflected in the marker size, which has been used to weight the fitting of the cumulative distribution function.

From a risk-benefit perspective, the risks in selection of the SI are the following: (1) too low SI will not activate the cortex that contains motor functions in mapped areas (and, as a result, false-negative responses are gained), and (2) too high SI that excites neurons outside the stimulated target region (resulting in a response that is falsely positive, i.e., positive responses not associated with the stimulated target). Provided that the fluctuation in cortical excitability is normal, and muscles are maintained in rest, the benefits corresponding with the above-mentioned risks are that with (1) low SI if the stimulation of a cortical target is producing a response, it can be assumed with high confidence as a true-positive response, and with (2) high SI the probability of inducing a response is greater (Figure 5), and it is likely that if there is no response the stimulated target is a true-negative response. Therefore, in selecting the SI, the risks and benefits should be weighted regarding the information that is needed to be acquired. In preoperative motor mapping in patients with brain tumors, a motor map that only shows a minimum number of false-positive stimulation points is warranted in order to avoid overestimation of the extent of the motor map. Such overestimation could lead to incomplete tumor resection given that false-positive spots are unnecessarily spared from resection due to anticipated, but faulty “true” motor function representations. However, high fractions of false-negative points would put the patient at a theoretically higher risk of functional deterioration in cases in which such points are included in the surgical resection. Correspondingly, Thordstein et al. speculated that using a low SI could include an additional risk since the activation area of a muscle could be distributed non-continuously along the motor cortex [107,120].

**Figure 6:**
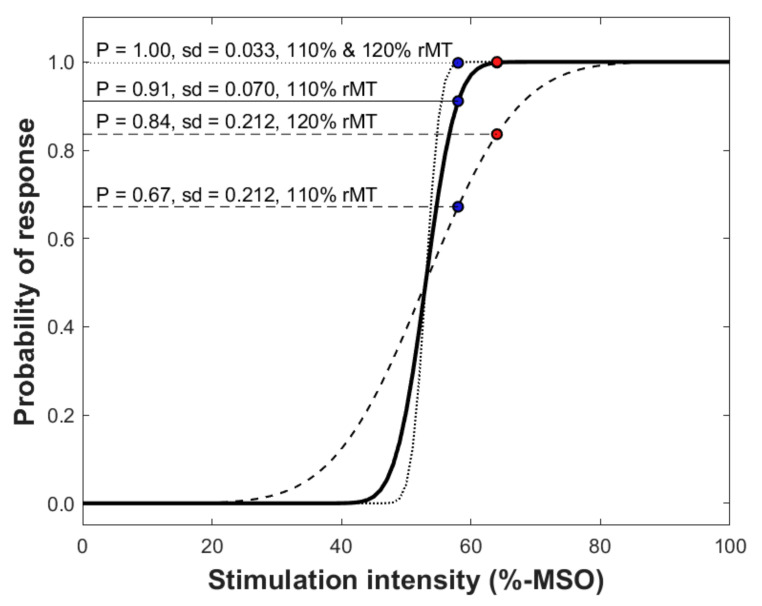
Demonstration of the cumulative distribution function representing the probability of inducing a response at different SIs. As the slope, represented as relative spread [110], can differ between subjects, the use of the SI related to the rMT can induce differences to the absolute size of the motor map [25]. In the plot, mean relative spread (*solid black line*) and the minimum (*dotted black line*) and maximum (*dashed black line*) found in the study population were used to compare probabilities of inducing a response at 110% rMT (*blue dots*) and 120% rMT (*red dots*). At a low relative spread, 110% and 120% rMT produce an MEP at the stimulation target with a probability close to 100%, but with the high relative spread, the probabilities of MEPs at the target at 110% and 120% rMT are 68% and 83%, respectively.

When using a SI that is at supra-threshold level normalized to the rMT (e.g., 110% rMT), it is unclear what the individual likelihood for induction of a response is (Figure 6). However, it may largely avoid false-negative stimulation points within the motor map by arbitrarily creating some sort of a “safety margin” around unequivocally motor-positive stimulation spots. Certainly, the likelihood for producing a response at the motor hotspot where the rMT was determined is greater than 50%—but is it 60% or 95%? This dilemma makes it difficult to estimate the real accuracy of individual motor mapping if the input–output characteristics are unknown. Alternative techniques for determining the mapping intensity based on a threshold value that relies on greater probability of responses than 50% has been suggested as an alternative way to determine the SI [25]. Specifically, Kallioniemi and Julkunen proposed that the use of the so-called Mills–Nithi UT could be used directly as the SI for mapping, and demonstrated that it indeed reduces the inter-individual variation in the quantified motor map size [25,108]. The core principle in using the UT instead of a SI normalized to the rMT seems justified as with UT the probability of a response is ~90%, hence reducing the dependence of mapping outcome from the individual input–output characteristics. However, there are drawbacks in that methodology: (1) the confidence in the estimated UT is likely lower than for the rMT that is determined using ATH [109,110], and (2) it may take a few minutes more time to determine the UT [25].

**Figure 7:**
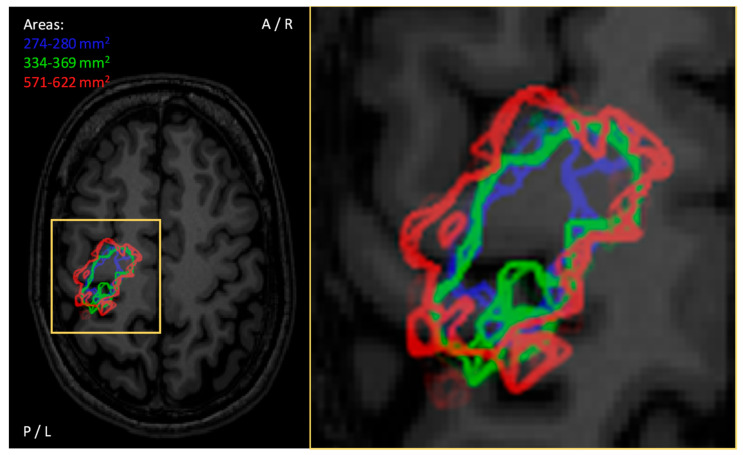
Visualization of the motor map resulting from mapping with three SIs (*blue*: 110% of the rMT, *green*: Mills–Nithi UT method [108], *red*: 120% rMT). Data from each of the experiments were bootstrapped 1000 times, assuming that 10% of the responses observed were false negative. The image is visualized in neurological projection. The stimulations were placed on average 0.4 mm apart. The 95% confidence limits are indicated for quantified areas in the images.

To demonstrate the variability of motor maps due to uncertainty in response occurrence probability, we simulated motor maps based on experimental motor mapping data by assuming that 10% of the responses were falsely negative to estimate the general confidence of motor mapping based on uncertainties related to response occurrence (Figure 7). The simulation demonstrated that despite the uncertainty, the SI-dependent differences in the motor map area were still apparent, and the shape and extent of the motor maps were maintained from simulation to simulation. Unlike the area of the motor map, the location of CoGs does not appear to depend on the SI [24]. It was demonstrated by Thickbroom et al. that when moving the coil from one cortical location to another, the shape of the input–output curve does not change significantly, only the offset that is the crucial part being represented as the rMT [121].

The SI is commonly represented as a percentage of the maximum stimulator output (%-MSO), by definition making it dependent on the maximal stimulator performance that is highly dependent on the used instrumentation including the characteristics of stimulation coils and the stimulating pulse [122,123,124,125,126,127,128]. This means that when comparing the used SIs between individuals, one has to consider the characteristics of the instrumentation. In addition, the SI, when represented as %-MSO, is not considering the individual distance of the stimulated cortex from the stimulating coil that is placed on the scalp [129,130,131,132,133]. To account for individual coil-to-cortex distance by estimating the cortically-induced EF by stimulation, an EF estimate could be used [134,135,136]. However, the EF estimate may not account for differences in pulse characteristics [137]; yet it could reduce the difference in representing SIs as EFs between stimulator manufactures differ [126]. Nevertheless, when applying EFs in different individuals, there exists a challenge to determine the anatomic location where the EF should be estimated, as the exact location of response induction has not been unambiguously determined since the activation by stimulation is not limited to the crown of specific layers of the cortex, but, instead, the coil distance-dependent EF affects a large part of the cortex [29,105,138].

Recently, Nazarova et al. mapped the representations of multiple muscles simultaneously to distinguish between muscle representations of the individual muscles [98]. As previously suggested, they observed that the use of a single SI may be a possible limitation as the different muscles could potentially have different rMTs [98]. Previously, it has been observed that the different somatotopically adjacent muscle representations could have different excitability profiles [95,107,118]. Albeit, at the group level the effect size may be minor or acceptable, at the individual level the clinical significance for such different profiles may be crucial [95,119,139,140,141]. This means that if a muscle has a lower rMT for activation and a steeper rise for the input–output curve than the other mapped muscles, the motor map will be biased due to the responses of that muscle, and will mostly present the representation area of that specific muscle over the other mapped muscles. Therefore, when determining quantitative characteristics for a group of muscles, the mapped outcome may be biased with certain muscles due to the differences in the individual muscle rMTs, and perhaps also due to individual motor hotspots.

Furthermore, the coil-to-cortex distance varies with stimulated cortical regions, which may require adjustment of the applied SI [130,131,135,136,142,143,144]. Because of the differences between target sites, the SI needs to be adjusted by taking into account the differences in coil-to-cortex distances, the secondary field caused by charge accumulation at conductivity discontinuities, and the coil orientation, and adjustment based only on the SI or primary EF is not sufficient [135].

### 3.3. Stimulation Grid

**Figure 8:**
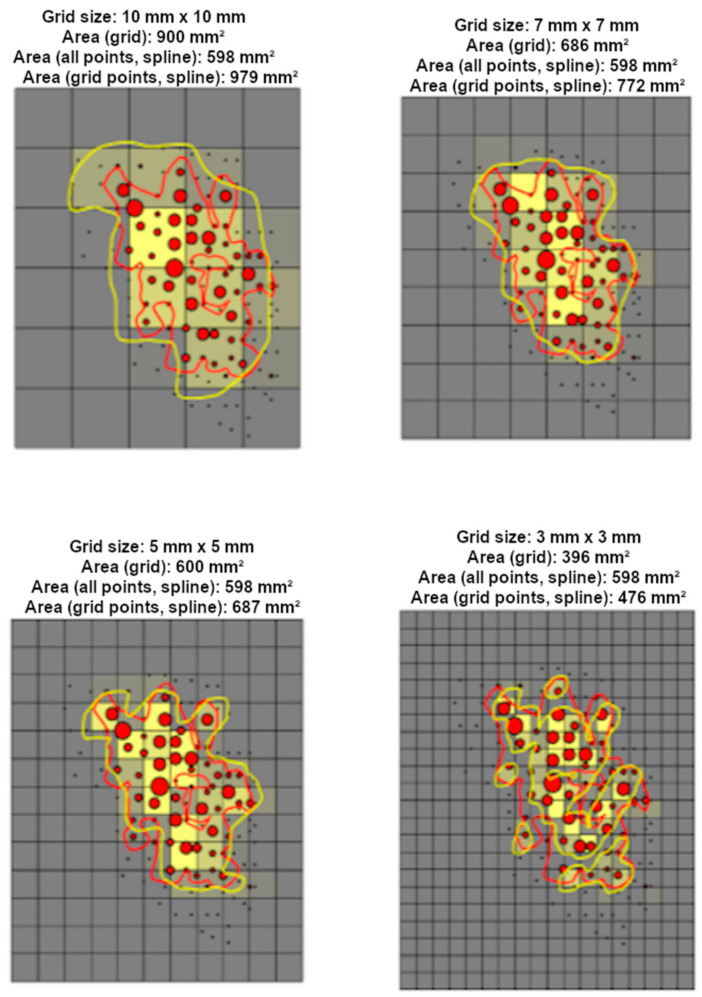
Demonstration of the use of a grid in calculating the area of the motor map. The original individual responses are presented as *red-filled dots*, with the dot size reflecting the MEP amplitude. The *black dots* are 0-amplitude responses. Average MEP amplitudes were calculated within grid squares in different size grids. The resulting average MEP amplitude size is reflected in the *yellow color* of the grid squares. The area of the motor map was evaluated based on the sum of the grid square areas with average MEP amplitudes of at least 50 µV, and by using spline interpolation (*yellow line*). The corresponding motor map areas are displayed above the grids with the grid sizes. For comparison, the motor map area is displayed for the original responses with spline interpolation, indicated by the *red line* in each plot.

**Figure 9:**
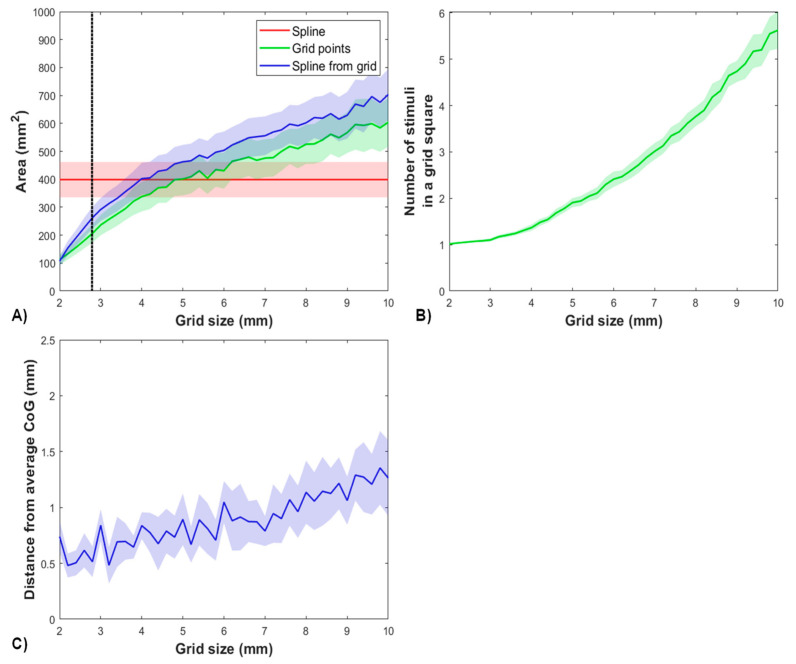
(**A**) The effect of grid size on the resulting motor areas with different methods of calculation. As a comparison, there is the area calculated with the spline interpolation (*red*), which is independent of the grid size, but is instead dependent on the local spacing of the stimuli. The area calculated from active grid squares is presented in *green* and, for ease of comparison, the spline interpolation area calculated from the active grid sites is shown in *blue*. The shaded areas indicate the 95% confidence interval within the study population, which was 24 mapping experiments at 110% of rMT. (**B**) The average number of stimuli falling within each grid square is shown as a function of grid size, with the shaded area indicating the 95% confidence interval within the study population. (**C**) The effect of grid size on the CoG location is shown, with the shaded area indicating the 95% confidence interval within the study population.

To enable quantitative mapping and to set the spatial density for stimulation targets and, thus, spatial accuracy of the quantitative mapping, stimulation grids are frequently used [27,145,146,147]. The grids are especially crucial for non-navigated estimation of motor maps [145,146]. However, they are also used in nTMS approaches where the underlying anatomy is visible [27,147]. The use of the grids enables straightforward calculation of the motor map size, i.e., by calculation of the number of active squares (producing acceptable responses when that square is stimulated) within the grid [118]. The definition of the active grid square varies in terms of interpreting a response (e.g., response/no response, maximum MEP amplitude, mean MEP amplitude, MEP count, etc.). In nTMS, the size of the grid squares affects the accuracy of the motor map that can be related to anatomical structures. The accuracy is limited also by the resolution of the underlying structural MRI and the accuracy of the neuronavigation system [1]. If using a grid as aid for enabling homogeneous spacing between the stimuli, the selection of the grid size should consider the required spatial resolution of the motor map. Figure 8 demonstrates how the size of a stimulation grid square could affect the appearance of a motor map. The larger grid squares result in bulk-shaped motor maps with the grid potentially overestimating the true map size, while reduction of the grid square size converges towards the true motor map size. However, the smallest grid squares produce lower map size than the reference value because the original data were gathered with inferior density and there might be no data for all grid squares (Figure 9).

It may soon be obsolete to consider motor mapping in terms of grids and grid targets, as modern nTMS motor mapping could potentially be performed in a quantitative fashion without the use of grids as long as quality criteria are set [148]. This means that the placement of stimuli is anatomically guided with denser placement of stimuli at specific regions, e.g., in the vicinity of anatomically interesting landmarks or at the edges of a motor map. This also means that the spatial and regional accuracy within the motor map may vary, while the extent of the motor map could be more accurately defined. The effects could be inverse for the accuracy of the CoGs, and for calculation of CoGs the coverage of each individual stimulus target in the motor map needs to be accounted for and be weighted in the calculation [27].

### 3.4. Number of Stimuli Required (per Target Location)

The accuracy of the motor mapping has been shown to relate to the number of stimuli used [26]. The number of stimulated responses within each grid square varies, as does the size of the grid squares [26,118,140,145,147,149,150,151,152,153]. Previous studies have investigated the required number of stimuli in a stimulation grid, having observed that two or more stimuli should be used to improve the confidence in the resulting motor map parameters [26,154]. Specifically, Cavaleri et al. reported that at least two responses induced by targeting each grid square were required for reliable calculation of the CoG and motor map volume in non-navigated TMS with a grid square size of 10 mm [154]. The used stimulus grid, or the density of the stimulus target spacing plays an essential role when defining the parameters of the motor map. In essence, the CoG location is dependent on the grid square size, as it is the case with the cortical area (Figure 9). The larger the grid square is, the less accurate is the CoG location or the motor area (Figure 9).

**Figure 10:**
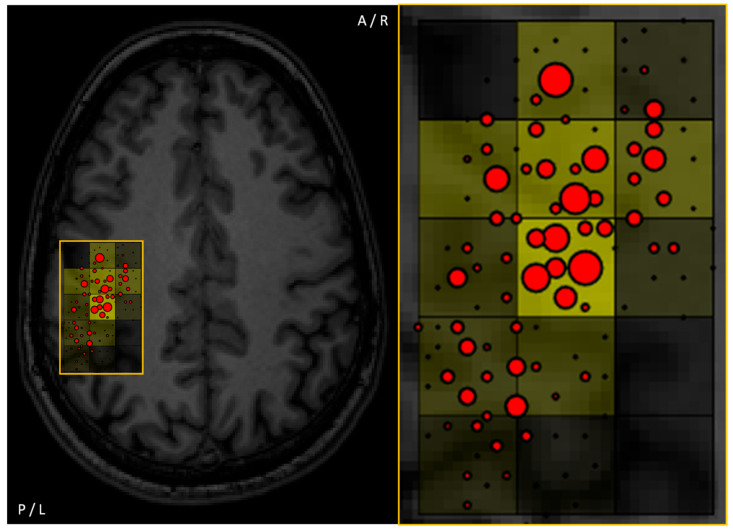
The visualized grid square size used was 10 mm × 10 mm, which is a quite commonly used size [146,149]. The stimulations were placed on average 2.6 mm apart. The individual stimulus locations are shown with *red dots*, the size of which is indicative of the associated MEP amplitude (the larger the amplitude, the larger the dot). The *yellow colors* in the grid squares indicate the size of the mean MEP amplitude for the MEPs induced by stimulating points within the grid square (the brighter the color, the higher the mean MEP amplitude).

When using nTMS, the conventional grid squares (e.g., 10 × 10 mm or 15 × 15 mm in size) are likely to include more stimuli. This is demonstrated in Figure 10, placing a stimulus grid of typical size over the mapped region and demonstrating that several stimuli are placed within the grid squares [146,149]. In fact, due to the spatial averaging caused by the large number of MEPs recorded during the mapping, the inherent variability effect in MEPs may be reduced, and placing multiple stimuli per location is compensated by closer spacing of the stimulus locations [95,155]. Chernyavskiy et al. showed that with an increasing number of stimuli included in the motor map, the accuracy is improved in nTMS mapping without application of a grid, as the coverage of a single response in a motor map and, hence, the contribution a response for the total motor map is decreased [155].

Often, when utilizing stimulation grids, a few stimuli may be repeated per grid to reduce the effect of MEP amplitude variation. However, then the number of stimuli is in a different scale than the number of required stimuli needed to obtain the average MEP amplitude confidently. To reach for a reliable and stable value of the MEP amplitude, previous studies have found that at least 20 repeated trials should be averaged [156,157]. However, these confidences are not fully comparable, since the overall data on MEP amplitudes within the motor maps are more extensive than in studies assessing single-target MEP amplitudes. Thus, the effect of individual grid square MEP amplitudes variability affects less the binarized map parameters such as the area, and, hence, the “spatial filtering” reduces the apparent MEP variability. Obviously, parameters utilizing absolute MEP amplitudes such as the map volume could be more affected.

### 3.5. Coil Orientation with Respect to Anatomy

The degrees of freedom (DOF) of the stimulation coil include the coil location, orientation and tilt, while additionally, one system-dependent DOF is also the previously discussed SI. The coil tilt affects the efficiency of dose delivery on the cortex [158,159]. With respect to the underlying cortical anatomy, the coil orientation affects the observed response (i.e., suboptimal coil orientation may result in MEPs with low amplitude or a non-response) [105,160,161,162]. Balslev et al. showed with non-navigated TMS that a 45° angle with respect to the interhemispheric midline is generally the optimal coil orientation [163]. Further, the optimal coil orientation has been observed from experiments and simulations to be perpendicular to the gyral wall [104,105,135,138,160,164]. While there may not be group-level differences in the optimal coil orientation, the individually quantified parameters, such as the motor area, may depend on it [106].

The microanatomy of the gyrus may also affect the efficacy of nTMS, i.e., how aligned are the stimulations and the activated neuronal structures, and how organized or anisotropic are the activated individual neurons in their population [138,165]. While these factors cannot be directly visualized during application of nTMS, they may take relevant effect on the mapping outcome.

### 3.6. Other Quantitative Parameters in Motor Maps

Other quantitative parameters commonly used to characterize motor maps based on MEP amplitudes include the motor hotspot, CoG, motor area, and motor map volume. The motor hotspot location typically is defined by the location of the maximum MEP amplitude response (*x_max_*; *y_max_*) or the “optimal location” for stimulation [28,29,166,167,168]. The motor hotspot is not only used to characterize the location of the motor representation, but is also used as the location where the rMT is determined and around which the motor mapping is extended [169]. Considering the definition of the motor hotspot, Reijonen et al. characterized a hotspot as a region instead of a unique target and found that if the definition is based on MEP amplitudes within individualized motor maps, the hotspot is on average 13 mm^2^ in size, and if the hotspot is defined on the basis of the stimulation-induced EF, the size is on average 26 mm^2^ [29]. These hotspot definitions consider the accuracy of the definition of the hotspot, including within-session neuronavigation system accuracy. Specifically, with nTMS, it has been shown that intra- and inter-observer variability for motor hotspot determination are on average ≤1 cm, with values ranging within the calculated precision of the used system [170].

**Figure 11:**
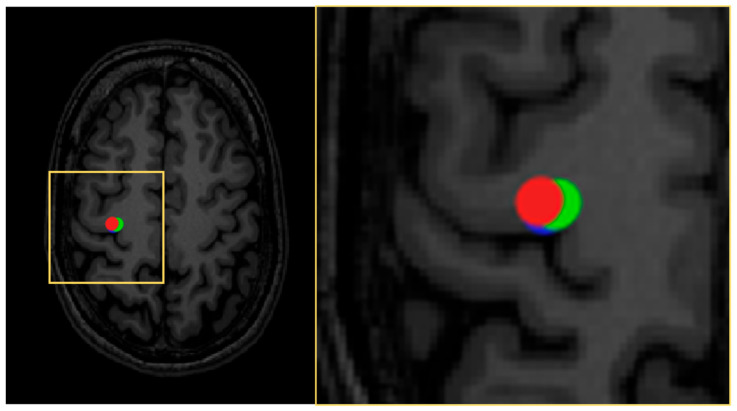
Visualization of CoGs based on motor maps with three SIs (blue: 110% of the rMT, green: Mills–Nithi UT method [108], red: 120% rMT). Data from each of the experiments were bootstrapped 1000 times, assuming that 10% of the responses observed were false negative. The dots indicate a product of 1000 CoG locations each. The image is visualized in neurological projection.

The CoG is defined as the amplitude-weighted location in coordinates representing the location in the motor representation area where the center of motor activation lies, and is represented by the following equation:

*x*_CoG_ = ∑*M_i_x_i_*/∑*M_i_*; *y*_CoG_ = ∑*M_i_y_i_*/∑*M_i_* (1)

where CoG location is defined in two dimensions with the Cartesian coordinates *x*_CoG_ and *y*_CoG_, individual MEP amplitudes, and *M_i_* corresponding to single stimulation targets (*x_i_*; *y_i_*) [171]. The CoG locations are dependent on the muscle representation mapped [95,118,172]. The inter-session repeatability of the CoG has been demonstrated to be good to excellent [28,31,98,118]. In our simulations, we found that the SI has a minor effect on the CoG location, as does the chance of false-negative responses (Figure 11).

If the spacing of the stimulus targets is not homogeneous within the motor map (i.e., when a stimulus grid is not used), then the response amplitudes *M_i_* need to be weighted by their stimulus targets coverage in the motor map *A_i_* [27]:

*x*_CoG_ = ∑*M_i_A_i_x_i_*/∑*M_i_A_i_*; *y*_CoG_ = ∑*M_i_A_i_y_i_*/∑*M_i_A_i_* (2)

The volume of a motor map is usually defined in a grid by summing the MEP amplitudes associated with the grid squares that exceed a given response threshold [24,30,31]:

*Volume* = ∑*M_i_* (3)

where the index *i* refers to each grid element. Alternatively, the volume of the motor map has been determined as the volume of an interpolated amplitude surface on the cortex [96]. The repeatability of the motor map volumes has been demonstrated to be between poor and good [30,31].

**Figure 12:**
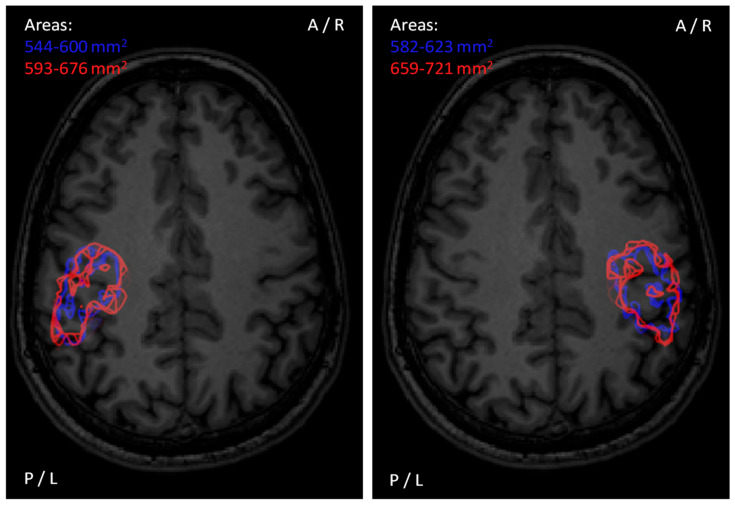
Visualization of motor map outlines of the FDI muscle representation in repeated measurements. Data from each of the experiments were bootstrapped 1000 times, assuming that 10% of the responses observed were false negative. The image is visualized in neurological projection. The *blue maps* were mapped a bit more densely (mean distance between stimulus locations was 2.6 mm on both hemispheres, as a 3.0 mm grid was used as aid) than the *red maps* (mean distance between stimulus locations was 2.7 mm and 3.0 mm on the left and right hemisphere, respectively, without a stimulation grid). The 95% confidence limits are indicated for quantified areas in the images.

**Figure 13:**
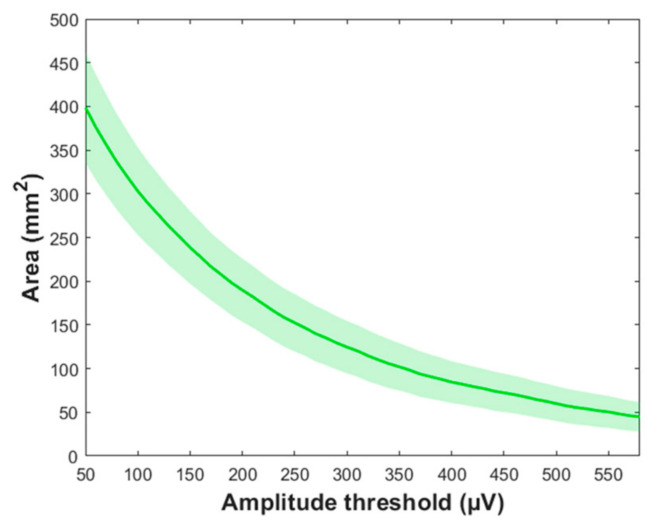
Effect of amplitude criterion for accepted MEPs measured on the resulting motor map size as determined using spline interpolation [27]. In the figure, 24 cortical mapping experiments for locating and outlining the FDI muscle representation were used to calculate the mean (*green line*) and 95% confidence interval (*green area*) for the motor area at different amplitude threshold criteria for accepted MEPs.

The area has been defined in different ways. When using a simulation grid, previous studies have multiplied the grid square area with the number of active stimulation sites within the grid [118,147,173]. With nTMS, recent studies have utilized different means for calculating the area, such as spline interpolation or Voronoi tessellation [25,27,95,153,155,173,174,175,176]. These analysis techniques of the representation are not directly comparable, as they include systematic differences [173]. Previous studies have shown that the motor map area may suffer from poor to excellent session-to-session/within-session repeatability [27,30,31,98,118]. Here, we simulated the session-to-session repeatability of a motor map by assuming the potential of false-negative motor responses to find that the quantitative size is well-preserved within the sessions as are the shapes and locations, while minor session-to-session differences were observed in the motor map area (Figure 12). The set amplitude criterion also affects the motor area, and commonly the amplitude criterion of 50 µV is used (Figure 13).

Sinitsyn et al. found, based on their experiments comparing multiple techniques and multiple stimuli/grid squares in calculating the motor map areas, that area weighted by the probability of an MEP within a grid square appeared overall best in terms of accuracy [26]. As the SI applied with respect to the rMT determines the probability of an MEP in each grid square, the reliable determination of the probability would likely require multiple repetitions per grid square, or for one stimulus per grid square the probability would be 0 or 1.

## 4. Perspectives and Future Directions

Over the past decade, nTMS has found its way into clinical routine, particularly for motor mapping among patients with motor-eloquent brain neoplasms since the method combines spatially resolved identification of brain function with high accuracy even in cases with deranged anatomo-functional architecture. The good agreement between preoperative nTMS motor mapping and intraoperative DES mapping as the reference method seems one of the key factors contributing to the current role of nTMS motor mapping in neuro-oncological surgery [35,36,37,38,39,40,41,42]. Furthermore, motor maps derived from nTMS can be used for seeding to achieve function-based tractography, which enables the identification of the spatial course of critical subcortical structures such as the CST [51,52,53,54,55,56]. However, the initial application for preoperative planning and intraoperative resection guidance in patients harboring functionally eloquent brain neoplasms has been greatly expanded over the years, thus enabling researchers to also address basic research questions in the context of a brain tumor as the use case, spanning from risk stratification for motor function to plasticity and reshaping of functional anatomy [69,70,71,72,73,77,78,79,80,81].

The key to successful integration of nTMS motor mapping and derived tractography into the clinical workflow is closely related to feasibility aspects and potential difficulties when embedding these approaches in existing environments. In this context, seamless integration into pre-existing infrastructure (e.g., hospital information system or PACS) can be achieved by standardized data export and transfer from the nTMS system [82]. However, it has to be noted that performing the mapping procedure in the most accurate fashion requires some training and time (~60 to 90 min per patient, excluding preparation and depending on factors such as the extent of motor mapping and patient cooperation) [6]. Thus, trained personnel and dedicated time slots for mapping purposes may be required in the clinical setting, which are not always granted under economic and time constraints. However, alternatives to nTMS motor mapping for the preoperative workup in patients harboring brain neoplasms (e.g., fMRI or MEG) also come with expenses and may only be available in specialized centers. Given that an overall better agreement between nTMS motor mapping and DES mapping has been observed in comparative studies with fMRI or MEG, efforts during the preoperative setup with nTMS seem justified [35,36,37,38,39,40,41,42].

Further potential of nTMS motor mapping lies in the interdisciplinary use of derived data. Particularly, radiotherapists may take advantage of such maps to modify treatment plans with the aim of limiting dose exposure to motor-eloquent cortex, as shown in first studies on the matter [83,84,85,86,87]. Integration of functional data derived from nTMS motor mapping could also be achieved for case discussions of interdisciplinary tumor boards and forwarded to follow-up treatment, which may make use of such information for rehabilitation strategies. In this context, a first example in patients suffering from acute surgery-related paresis of uE muscles after glioma resection provides evidence for the beneficial use of nTMS as a therapeutic tool in neuro-oncology, with the exact site of stimulation being determined based on nTMS motor maps [177]. Specifically, the combination of low-frequency nTMS with physical therapy for seven consecutive days after surgery improved motor function outcome according to the Fugl–Meyer assessment performed postoperatively and until the 3-months follow-up examinations [177].

Additionally, nTMS data could also be efficiently used in multi-modal scenarios. For instance, the combination of motor mapping with mapping of other functions such as language and derived tractography of motor- and language-related subcortical pathways has already been achieved in a few studies, which may help to gain a broader picture of functional anatomy in patients harboring large or critically situated neoplasms that most likely do not solely affect the motor system [178,179]. Further, to understand better the distinct effects of nTMS on the brain’s connectivity profile, combinations of nTMS application with pre- and post-stimulation fMRI acquisitions and functional connectivity analyses are possible and have shown promising results in healthy volunteers [100,101]. Most notably, it has been revealed that modulation by nTMS critically depends on the connectivity profile of the target region, with imaging biomarkers derived from fMRI possibly playing a role to improve sensitivity of nTMS for research and clinical applications [100]. Based on such data, it seems likely that in patients with brain neoplasms, the impact and effects of nTMS also depend on a connectivity profile that may fluctuate over time or due to yet unidentified parameters, possibly interfering with the mapping outcome. Yet, multi-modal scenarios specifically in patients with motor-eloquent brain tumors may also exert difficulties on data acquisition, processing, and interpretation of data, which need to be addressed prior to routine application. For fMRI, presence of particularly high-grade tumors with increased cerebral blood flow characteristics can negatively interfere with signal interpretation [180,181]. Thus, combinations of fMRI with nTMS in such patients need to be considered carefully to avoid errors in calculations of connectivity characteristics.

Regarding dMRI, images can suffer from geometric image distortions compared with anatomical MRI, which may introduce spatial inaccuracies when dMRI data is linearly projected on conventional T1- or T2-weighted sequences and used for tractography. This may be retrospectively corrected for by non-linear, semi-elastic image fusion, thus potentially enabling tractography with improved accuracy and clinical feasibility [182]. Expanding on this, intraoperative MRI-based elastic image fusion for anatomically accurate tractography of the CST using nTMS motor maps has been achieved, correlating well with IONM and disproving the severity of brain shift in selected cases [183,184]. Furthermore, most work on nTMS-based fiber tracking of the CST has used standard deterministic algorithms implemented in commercially available packages for clinical use (e.g., fiber assignment by continuous tracking (FACT) algorithms) [51,52,54,55,70,71,72]. Using a FACT algorithm, fiber bundles are reconstructed in a voxel-by-voxel fashion with respect to the direction of the main eigenvector, which works purely data-based (no interpolation function) and needs only comparatively low computation time [185,186]. However, on the other hand, FACT algorithms create some predictable inherent errors, which limit the accuracy of the method and could lead to error-prone or incomplete tractography results [186,187,188,189]. Probabilistic approaches disperse trajectories more than deterministic methods and may delineate a greater proportion of white matter tracts, particularly when combined with more advanced dMRI sequences [189]. Additionally, the potential value of diffusion measures besides mere delineation of the spatial course of the CST by nTMS-based tractography may be of merit. One study has shown that the extent of impairment of diffusion metrics (such as FA and ADC) correlates with motor function deficits according to segmental analyses within the CST [72]. Hence, supplementing nTMS-based tractography of the CST with diffusion metrics may improve the predictive power for postoperative motor impairment, but other parameters such as mean diffusivity (MD) have not yet been routinely considered. As such, MD is a quantitative measure of the mean motion of water and reflects the rotationally invariant magnitude of water diffusion, which could also be representative of structural integrity of white matter [190]. Future work may explore the potential benefits of further, even more elaborate quantitative markers of white matter structure, composition, and integrity, which can be derived from advanced dMRI techniques such as high angular resolution diffusion imaging, multi-shell imaging, diffusion kurtosis imaging, neurite orientation dispersion, and density imaging [191,192,193].

The continuous optimization of the nTMS system technique has recently enabled a novel paired-pulse nTMS (pp-nTMS) paradigm for biphasic pulse wave application to induce short-interval intracortical facilitation [194,195,196]. Use of pp-nTMS may increase efficacy of motor mapping in patients with brain tumors as accurate motor maps are achieved even in cases where conventional single-pulse mapping fails (e.g., due to tumor-affected motor structures or edema) [174,175]. Further, pp-nTMS would result in a lower rMT, thus allowing motor mapping with lower SI but without clinically relevant constraints for motor map extent or location [174,175]. Particularly in patients with brain tumors, the rMT can be frequently high in the tumor-affected hemisphere, and accurate mapping with a lower SI related to a lower determined rMT could permit successful use of nTMS even in the most demanding cases. This may also be highly relevant for other applications of nTMS in patients with brain tumors, such as language mapping [197,198,199,200,201]. In such applications, a higher SI is often used and stimulation is more widespread and, thus, can entail discomfort that negatively interferes with the mapping outcome. However, studies using pp-nTMS for other purposes than motor mapping are currently lacking.

**Figure 14:**
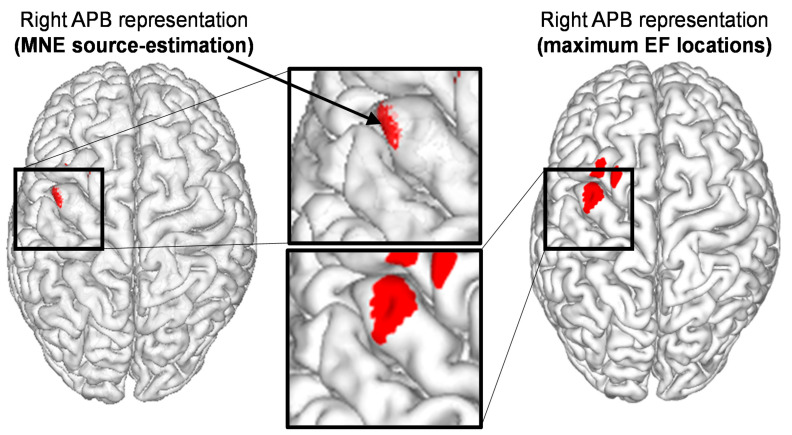
The MNE method (**left**) utilizes the spread of the EF at each stimulation point in addition to the MEP amplitude to estimate the source of motor activation, as opposed to the more conventional motor mapping approach not considering the spread of the EF associated with each stimulus (**right**) [204]. The active stimulation sites in the conventional motor map were outlined using the spline interpolation method [27].

From a methodological perspective, the presently used motor mapping approach is focused on determining the volume, area, coil locations, or corresponding cortical EF maximum locations associated with motor responses. However, as the EF induced by nTMS is not ideally focused, the spread of the EF also stimulates adjacent areas and, thus, when stimulating an area in the cortex adjacent to the target muscle representation area, the target muscle may activate even though the representation area was not targeted. Hence, quantitative mapping at present cannot precisely capture the true representation size of the motor representation area, and may over- or underestimate it. Previous studies have shown that inclusion of the EF information in generating the motor maps may aid in localizing the motor representation [202,203,204]. Specifically, minimum norm estimation (MNE) has recently been employed for estimating the true representation area of muscles by accounting for the EF spread and the input–output characteristics of the MEP values [204]. Figure 14 demonstrates the application of MNE in a single subject in comparison with the outlined cortical maximum EF locations with associated positive responses. A quite similar method to MNE was presented by Weise et al., also utilizing the input–output characteristics of the MEPs [203]. The practical difference between these methods arises from the number of coil locations applied and how the input–output characteristics are estimated spatially.

In essence, one challenge for more accurate clinical motor mapping with nTMS in the future may be the accurate inclusion of the EF information to estimate the source of the induced MEPs. Currently, there are no clinically validated tools classified as medical devices available for source estimation utilizing EF spread, and the tools that are currently available require special skills and are not feasible for clinical routines. However, the development seems to be heading in the right direction. With tools such as the SimNIBS, EF simulations have been made quite straightforward, albeit the pipelines feasible for clinical mapping applications are still lacking [205,206]. Furthermore, the shape of the motor map may be evaluated by the aspect ratio (i.e., the ratio between map extension along the EF direction and perpendicular to it) [195]. This means that if the aspect ratio is 1, the shape of the motor map is approximated as circular, if the ratio is >1, the map is elongated along the EF direction, and with an aspect ratio <1, the motor map is elongated to the direction perpendicular to the EF direction. With single-pulse nTMS, the aspect ratio tends to be >1 [174,175,195].

Despite increasing acceptance of nTMS motor mapping in clinical routine, it cannot be emphasized enough that the value of the method stands and falls with its accuracy. In this regard, many parameters such as the location of the motor hotspot and CoG, and area and volume of the motor map are associated with the applied SI, which needs to be determined with highest diligence. Therefore, with the increase in applications of nTMS, methods for ensuring feasible accuracy become more and more important. In the context of quantitative mapping, awareness of the relevant parameters and control over them is warranted to assure best practices and reliable mapping outcomes. Specifically, quantitative mapping has the potential to derive parameters related to the motor maps of the patients that could impact diagnostics, prognostics, and follow-up examinations by enabling spatial and spatio-temporal metrics related to cortical motor function. Major future development efforts should be put to understanding the correlations between motor mapping, interpretation of stimulation targets, and resulting responses in relation to their origin in the cortex by consideration of the physical effects of induced EFs.

## 5. Conclusions

The technique of nTMS is increasingly used particularly for preoperative motor mapping in patients harboring brain tumors, which is due to its sufficient accuracy and reliability in a clinical setup. The combination of nTMS motor mapping with tractography as well as the option of serial mapping over time profoundly expands its role beyond a mere surgical planning tool. Development of quantitative motor mapping can include further applications while the accuracy of current mapping modalities can be improved by standardized protocols and increased consideration of EF information.

## Figures and Tables

**Table 1:** Feasibility, reliability, and comparison with other methods. This table outlines the studies published on feasibility and reliability of motor mapping by nTMS (using an electric-field-navigated system) in patients harboring brain neoplasms. Furthermore, studies comparing nTMS motor mapping with other modalities (intraoperative DES, preoperative fMRI, and preoperative MEG) are included.

Author	Year	Cohort	Tumor Entities	nTMS Method	Techniques for Comparison	Main Objective	Main Findings
Coburger et al. [32]	2011	75-year-old woman	MEN	Preoperative motor mapping (60–80 V/m)	Intraoperative DES	To correlate nTMS with DES	− The motor strip as determined by DES was located in the same cortical area where motor responses were elicited by nTMS. − nTMS motor mapping is a helpful tool for preoperative planning.
Picht et al. [33]	2011	63-year-old woman	MET	Preoperative and postoperative (4th postoperative day) motor mapping (110% rMT)	Intraoperative DES	To test nTMS in a patient with severe preoperative motor impairment and to correlate with DES	− nTMS motor mapping demonstrated intact corticospinal pathways in presence of hemiplegia. − nTMS motor mapping modified the surgical strategy. − nTMS and DES mappings agreed well.
Picht et al. [35]	2011	20 adult patients	HGG, MET, MEN, Other	Preoperative motor mapping (110% rMT)	Intraoperative DES	To compare the accuracy of nTMS with DES	− Motor hotspots were located on the same gyrus for nTMS and DES mappings in all cases. − Distances between motor hotspots of nTMS and DES mappings amounted to 7.8 ± 1.2 mm for the APB and to 7.1 ± 0.9 mm for the TA muscles. − nTMS and DES mappings agreed well.
Forster et al. [37]	2011	11 adult patients	LGG, HGG, MET, Other	Preoperative motor mapping (110% rMT)	Preoperative fMRI Intraoperative DES	To compare the accuracy of nTMS with fMRI and DES	− Distances between motor hotspots of nTMS and DES mappings (10.5 ± 5.7 mm) were significantly smaller than those between the activation in fMRI and DES motor hotspots (15.0 ± 7.6 mm). − nTMS motor mapping is more precise than fMRI when correlated to results of DES.
Picht et al. [43]	2012	73 adult patients	LGG, HGG, MET, MEN, Other	Preoperative motor mapping (110% rMT) and nTMS-based tractography (but not considered)	Intraoperative DES	To evaluate the influence, benefit, and impact of nTMS on surgery	− nTMS motor mapping confirmed the expected functional anatomy in 22%, added awareness of high-risk areas in 27%, modified the approach in 16%, changed the planned EOR in 8%, and changed the surgical indication in 3% of patients. − nTMS motor mapping has positive impact on surgical planning and on the surgery itself.
Krieg et al. [38]	2012	26 adult patients	LGG, HGG, MET, Other	Preoperative motor mapping (110% rMT for uE and 130% for lE muscles)	Preoperative fMRI Intraoperative DES	To compare the accuracy of nTMS with fMRI and DES	− Distances between motor hotspots of nTMS and DES mappings (4.4 ± 3.4 mm) were significantly smaller than those between the activation in fMRI and nTMS motor hotspots (9.8 ± 8.5 mm for uE and 14.7 ± 12.4 mm for lE muscles). − In most cases of tumors in the precentral gyrus the neurosurgeon admitted easier identification of the central region in awareness of nTMS motor maps. − nTMS and DES mappings agreed well.
Coburger et al. [34]	2012	3-year-old boy	LGG	Preoperative motor mapping (130 V/m for uE and 220 V/m for lE muscles)	Intraoperative DES	To test nTMS in a child and to correlate with DES	− DES verified the location of nTMS motor hotspots. − nTMS is a precise tool for preoperative motor mapping and is feasible even in young, pediatric patients.
Tarapore et al. [42]	2012	24 adult patients	LGG, HGG, Other	Preoperative motor mapping (110% rMT)	Preoperative MEG Intraoperative DES	To compare the accuracy of nTMS with MEG and DES	− Distances between motor hotspots of nTMS and DES mappings (2.1 ± 0.3 mm) were significantly smaller than those between the activation in MEG and nTMS motor hotspots (4.7 ± 1.1 mm). − DES did not reveal a motor site that was unrecognized by nTMS motor mapping in any patient. − nTMS motor mapping agreed well with DES as well as preoperative MEG.
Coburger et al. [36]	2013	28 adult patients and 2 patients <18 years	LGG, HGG, MET, MEN, Other	Preoperative motor mapping (110% rMT in adult patients and aMT in patients <18 years)	Preoperative fMRI Intraoperative DES	To compare the accuracy of nTMS with fMRI and DES	− Mean accuracy to localize the motor cortex was higher for nTMS motor mapping compared with fMRI. − In the subgroup of intrinsic tumors, nTMS motor mapping produced significantly higher accuracy for lE muscle representations than fMRI.
Krieg et al. [39]	2013	31 adult patients	LGG, HGG, MET, MEN, Other	Preoperative motor mapping (110% rMT for uE and 130% for lE muscles)	Preoperative fMRI Intraoperative DES	To compare the accuracy of nTMS with fMRI and DES in recurrent gliomas vs. initially operated tumors	− nTMS motor mapping correlated well with DES in recurrent gliomas (6.2 ± 6.0 mm) and newly diagnosed tumors (5.7 ± 4.6 mm), yet without a significant difference. − Compared with fMRI, the difference was larger for uE muscle representations (recurrent: 8.5 ± 7.2 mm; new: 9.8 ± 8.6 mm) and lE muscle representations (recurrent: 17.1 ± 10.6 mm; new: 13.8 ± 13.0 mm), yet without a significant difference. − nTMS motor mapping was as accurate in recurrent gliomas as it has been prior to the initial surgery.
Mangraviti et al. [40]	2013	8 adul tpatients	LGG, HGG, MET, Other	Preoperative motor mapping (110% rMT for uE and 130% for lE muscles)	Preoperative fMRI Intraoperative DES	To compare the accuracy of nTMS with fMRI and DES	− Distances between motor hotspots of nTMS and DES mappings (8.5 ± 4.6 mm) were significantly smaller than those between the activation in fMRI and DES motor hotspots (12.9 ± 5.7 mm). − Visualization of nTMS motor hotspots improved the neurosurgeons’ confidence in identifying the motor strip as well as in planning of surgical strategies.
Zdunczyk et al. [45]	2013	10 adult patients (and 10 healthy volunteers)	LGG, HGG, MET, MEN, Other	Preoperative motor mapping (110% rMT) (and two motor mappings per subject in the healthy volunteers)	-	To assess the intra- and inter-examiner reliability of nTMS mapping	− Distances between CoGs for the expert examiner in healthy volunteers were 4.4 (1.9–7.7) mm and 4.9 (2.4–9.2) mm for the expert vs. novice examiner. − The reliability of nTMS motor mapping in tumor patients appeared to be comparable to those in healthy subjects.
Rizzo et al. [44]	2014	17 adult patients	LGG, HGG, MET, MEN, Other	Preoperative motor mapping (110–115% rMT for uE and 130% for lE muscles)	Intraoperative DES	To evaluate the influence, benefit, and impact of nTMS on surgery	− nTMS motor mapping exactly localized the motor cortex in 88.2%, provided the neurosurgeon with new unexpected information about functional anatomy of the motor area in 70.6%, and led to a change of the surgical strategy in 29.4% of patients. − nTMS motor mapping has objective and subjective benefits on surgical planning and on the surgery itself.
Sollmann et al. [46]	2017	100 adult patients	LGG, HGG, MET, Other	Preoperative motor mapping (110% rMT for uE and 130% for lE muscles)	-	To investigate factors that have impact on MEP latencies	− Common factors (relevant to APB, ADM, and FCR) for MEP latency variability were sex and AED intake. − Muscle-specific factors (relevant to APB, ADM, or FCR) for MEP latency variability were tumor side, tumor location, and rMT.
Sollmann et al. [47]	2017	100 adult patients	LGG, HGG, MET, Other	Preoperative motor mapping (110% rMT for uE and 130% for lE muscles)	-	To investigate factors that influence the determination of the rMT	− Edema and age at exam in the ADM model only jointly reduced the unexplained variance for rMT determination. − The other factors kept in the ADM model (sex, AED intake, and motor deficit) and each of the factors kept in the APB and FCR models independently and significantly reduced the unexplained variance for rMT determination.
Lam et al. [49]	2019	20 adult patients	n/a	Preoperative motor mapping (105% rMT)	-	To investigate the feasibility of increasing the MEP threshold (50 μV vs. 500 μV) to improve the robustness of motor mapping	− Both the standard (50 μV) as well as the experimental (500 μV) MEP threshold yielded motor maps in all patients. − No significant differences in motor area sizes were found between the conventional (50 μV) MEP threshold and the experimental (500 μV) MEP threshold. − MEP latency time was significantly reduced for recordings from 500 μV compared with recordings from 50 μV MEP thresholds.
Weiss-Lucas et al. [41]	2020	36 adult patients	LGG, HGG, MET, MEN, Others	Preoperative motor mapping (110% rMT)	Preoperative fMRI Intraoperative DES	To compare the accuracy of nTMS with fMRI and DES	− There were significantly smaller Euclidean distances (11.4 ± 8.3 vs. 16.8 ± 7.0 mm) and better spatial overlaps (64 ± 38% vs. 37 ± 37%) between DES and nTMS mappings compared with DES vs. fMRI. − Contrary to DES, fMRI and nTMS motor mappings were feasible for all regions and patients without complications (reliable and accurate DES was only obtained in 25 of the included patients).
Mirbagheri et al. [50]	2020	12 adult patients (and six healthy volunteers)	n/a	Preoperative motor mapping (105% rMT for primary motor areas, 120%/150% for non-primary motor areas)	-	To investigate whether nTMS reliably elicits MEPs outside of the primary motor cortex	− 88.8% of stimulations in suspected non-primary motor areas did not result in motor-positive spots with MEPs ≥50 μV. − Positive nTMS motor mapping in non-primary motor areas was associated with higher SI and larger primary motor areas. − Particularly when mapped with 150% rMT, more MEP artifacts occurred in patients than in healthy volunteers.
Lavrador et al. [48]	2020	45 adult patients	LGG, HGG	Preoperative motor mapping (105% rMT)	-	To assess the excitability of the motor system in relation to tumor grading	− MEP latencies of lE muscles increased with an increase in the WHO grading of the tumor. − An association between the increase in the WHO grading and a decreased rMT was observed for lE muscles. − Higher WHO grading of the tumor and isocitrate dehydrogenase wild-type tumors were associated with the number of abnormal interhemispheric rMT ratios.

Abbreviations: nTMS—navigated transcranial magnetic stimulation; LGG—low-grade glioma; HGG—high-grade glioma; MET—metastasis; MEN—meningioma; rMT—resting motor threshold; aMT—active motor threshold; uE—upper extremity; lE—lower extremity; DES—direct electrical stimulation; fMRI—functional magnetic resonance imaging; APB—abductor pollicis brevis; TA—tibialis anterior; EOR—extent of resection; MEG—magnetoencephalography; CoG—center of gravity; ADM—abductor digiti minimi; FCR—flexor carpi radialis; MEP—motor-evoked potential; AED—antiepileptic drug; SI—stimulation intensity; WHO—World Health Organization.

**Table 2:** Fiber tractography. This table presents the studies published on tractography of the CST primarily using motor mapping by nTMS (using an electric-field-navigated system) for seeding in patients harboring brain neoplasms.

Author	Year	Cohort	Tumor Entities	dMRI Acquisition	Tractography Specifics	Main Objective	Main Findings
Krieg et al. [51]	2012	30 adult patients	LGG, HGG, MET, MEN, Other	6 diffusion directions, b-values: 0–800 s/mm^2^ (3 Tesla)	− Two ROIs: motor-positive nTMS points and ipsilateral brainstem (conventional: manually delineated motor cortex and ipsilateral brainstem) − Deterministic tracking: FA <0.2, FL ~100 mm, angular threshold of 30°	To assess the feasibility of nTMS-based tractography in relation to conventional seeding without nTMS data	− nTMS-based tractography resulted in a lower number of aberrant tracts (i.e., tracts not belonging to the CST) when compared with conventional seeding without nTMS. − The proximity of the tracts to the tumor was not different between nTMS-based and conventional tractography for CST reconstruction. − Conventional seeding showed to be user-dependent, whereas nTMS-based tractography seemed to be less subjective.
Frey et al. [52]	2012	50 adult patients	LGG, HGG, MET, MEN	23 diffusion directions, b-values: 0–1000 s/mm^2^ (3 Tesla)	− One ROI: motor-positive nTMS points (conventional: manually delineated internal capsule or brainstem) − Deterministic tracking: FA = 50% and 75% FAT, FL = 110 mm, angular threshold of 30°	To assess the feasibility and impact on surgery of nTMS-based tractography in relation to conventional seeding without nTMS data and to provide a new algorithm for FA determination	− nTMS-based tractography changed or modified the surgical strategy in 46% of patients, whereas conventional tractography would have changed the surgical strategy in only 22% of patients. − Tractography facilitated intraoperative situs orientation and application of DES in 56% of patients. − Tracking at 75% FAT was considered most beneficial by the neurosurgeons.
Conti et al. [53]	2014	20 adult patients	LGG, HGG, MET, Other	32 diffusion directions (3 Tesla)	− Two ROIs: motor-positive nTMS points, subdivided for uE, lE, and face muscles, and ipsilateral brainstem (conventional: ipsilateral brainstem) − Deterministic tracking: FA = 0.2, FL = 20 mm, angular threshold of 45°	To assess somatotopic organization by nTMS-based tractography in relation to conventional seeding without nTMS data and to verify nTMS-based tractography by intraoperative DES	− Detailed somatotopic CST reconstruction was possible by nTMS-based tractography with a greater overlap between the motor cortex and the cortical end-region of the CST (90.5 ± 8.8% vs. 58.3 ± 16.6%) when compared with conventional tractography. − DES mapping confirmed the CST location and the somatotopic reconstruction in all cases. − nTMS-based tractography of the CST appeared to be more accurate, less user-dependent, and capable of providing reliable CST delineation compared with conventional tractography.
Weiss et al. [54]	2015	32 adult patients	LGG, HGG, MET, MEN, Other	30 diffusion directions, b-values: 0–800 s/mm^2^ (3 Tesla)	− Two ROIs: motor-positive nTMS points, subdivided for uE, lE, and face muscles, and internal capsule and/or anterior inferior pontine level − Deterministic tracking: FA = 75% and 100% FAT, FL = 1 mm, angular threshold of 30°	To assess the impact of subcortical seed regions and of somatotopic location of cortical seed regions on plausibility of tractography in relation to clinical factors	− A higher proportion of plausible tracts was observed for seeding at the anterior inferior pontine level when compared with seeding at the internal capsule. − Low FAT and the presence of peritumoral edema within the internal capsule led to less plausible tractography, and most plausible tractography was obtained when the FAT ranged above a cut-off of 0.105. − A strong effect of somatotopic location of the seed region was observed, with the best plausibility present for tractography of fibers subserving the bilateral uE muscle representations (>95%).
Weiss et al. [55]	2017	18 adult patients	LGG, HGG, MET, MEN, Other	30 diffusion directions, b-values: 0–800 s/mm^2^ (3 Tesla)	− Two ROIs: motor-positive nTMS points, subdivided for uE, lE, and face muscles, and anterior inferior pontine level (fMRI-based: task-derived activation map and anterior inferior pons) − Deterministic tracking: FA = 100% FAT, FL = 1 mm, angular threshold of 30°	To assess the impact of the modality used for somatotopic location of cortical seed regions on plausibility of tractography	− A higher plausibility was observed for nTMS-based tractography compared with fMRI-based tractography, with fMRI-originated tracts showing a significantly more posterior course relative to the nTMS-based tracts. − nTMS motor mapping seems to be the method of choice to identify seed regions for tractography of the CST in patients with close vicinity of the primary motor cortex to a brain neoplasm.
Münnich et al. [56]	2019	11 adolescent or adult patients	HGG, MET, Other	20 diffusion directions, b-values: 0–700 s/mm^2^ (3 Tesla)	− Several ROIs for motor-positive nTMS points, task-derived fMRI activation maps, or conventional seeding − Deterministic and probabilistic tracking: minimal FA threshold = 0.05/∈ [0.1;0.45], tracking step length = 1 mm/∈ [0.5;7] mm, maximum fiber curvature = 0.3/∈ [0.2; 0.65]	To compare different seeding setups and tracking algorithms, and to correlate tractography with intraoperative DES and MRI findings	− The best accuracy of tractography was achieved using the segmented precentral gyrus for seeding (marginal R^2^ = 0.146); however, since the marginal R^2^ of fMRI and nTMS motor mapping differed very little, none of the methods showed distinct superiority. − Both nTMS-based and fMRI-based tractography showed significant correlations between distances and the SI of DES for the CST, but only with respect to uE muscle representations. − The use of the probabilistic tracking algorithm led to a better correlation between DES mapping and tractography. − Tractography demands for careful interpretation of its results by considering all influencing variables (e.g., seeding approach and tracking algorithm used).

Abbreviations: nTMS—navigated transcranial magnetic stimulation; LGG—low-grade glioma; HGG—high-grade glioma; MET—metastasis; MEN—meningioma; CST—corticospinal tract; dMRI—diffusion magnetic resonance imaging; ROI—region of interest; FA—fractional anisotropy; FAT—fractional anisotropy threshold; FL—fiber length; fMRI—functional magnetic resonance imaging; uE—upper extremity; lE—lower extremity; DES—direct electrical stimulation; SI—stimulation intensity.

**Table 3:** Improvement of clinical outcome. This table outlines the studies published on improvements of clinical outcome through the use of motor mapping by nTMS (using an electric-field-navigated system) with or without additional nTMS-based tractography in patients harboring brain neoplasms.

Author	Year	Cohort	Group for Comparison	Tumor Entities	nTMS Method	Outcome Parameters	Main Objective	Main Findings
Picht et al. [57]	2013	11 adult patients	11 historical controls	(suspected) LGG, HGG	Preoperative motor mapping	− Influence on surgery − EOR/tumor volume − Motor function (BMRC)	To assess the impact of nTMS on the treatment strategy and clinical outcome	− In 6 out of 11 patients, nTMS changed the treatment plan towards early and more extensive resection. − One of 4 patients of the nTMS group with preoperative motor deficits improved by one year, whereas increased motor deficits were observed in 3 of the 8 patients of the non-nTMS group not having surgery. − Median change of tumor volume from baseline to one year was −83% in the nTMS group and +12% in the non-nTMS group.
Frey et al. [58]	2014	250 adult patients	115 historical controls	LGG, HGG, MET, Other	Preoperative motor mapping and nTMS-based tractography	− Influence on surgery − EOR/tumor volume − Motor function (BMRC) − KPS − PFS	To assess the impact of nTMS on the treatment strategy, clinical outcome, and survival	− nTMS disproved suspected involvement of the primary motor cortex in 25.1%, expanded surgical indication in 14.8%, and led to planning of more extensive resections in 35.2% and more restrictive resections in 3.5% of patients. − The rate of GTR was significantly higher in the nTMS group (42% vs. 59%), and PFS for patients with LGG was better in the nTMS group (at 22.4 months) than in the non-nTMS group (at 15.4 months). − Integration of nTMS led to a non-significant change of postoperative deficits from 8.5% in the non-nTMS group to 6.1% in the nTMS group. − Expanding surgical indications and EOR based on nTMS might enable more patients to undergo surgery and could lead to better motor function outcome and improved PFS.
Krieg et al. [59]	2014	100 adult patients	100 historical controls (matching criteria: tumor location, preoperative paresis, and histology)	LGG, HGG, MET, Other	Preoperative motor mapping (110% rMT for uE and 130% for lE muscles) and nTMS-based tractography	− Influence on surgery − EOR/tumor volume − Motor function (BMRC)	To assess the impact of nTMS on the treatment strategy, clinical outcome, and survival	− Patients of the nTMS group showed significantly smaller craniotomies. − 12% of patients of the nTMS and 1% of patients of the non-nTMS group improved, while 13% and 18% of patients in the nTMS and non-nTMS groups, respectively, deteriorated in postoperative motor function on long-term follow-up. − Patients of the nTMS group showed a lower rate of residual tumor tissue according to postoperative MRI (odds ratio = 0.3828; 95% confidence interval = 0.2062–0.7107).
Krieg et al. [61]	2015	70 adult patients	70 historical controls (matching criteria: tumor location, preoperative paresis, and histology)	HGG	Preoperative motor mapping (110% rMT for uE and 130% for lE muscles) and nTMS-based tractography	− Influence on surgery − Perioperative complications − Adjuvant therapy − EOR/tumor volume − Motor function (BMRC) − KPS − PFS/overall survival	To assess the impact of nTMS on the treatment strategy, clinical outcome including direct perioperative complications, and survival	− Patients of the nTMS group showed significantly smaller craniotomies. − Residual tumor tissue (nTMS group: 34.3%, non-nTMS group: 54.3%) and unexpected tumor residuals (nTMS group: 15.7%, non-nTMS group: 32.9%) were significantly less frequent in the nTMS group. − Patients of the nTMS group were significantly more frequently eligible for postoperative radiotherapy (nTMS group: 67.1%, non-nTMS group: 48.6%). − 3-, 6-, and 9-month survival rates were significantly better in the nTMS group.
Picht et al. [62]	2016	93 adult patients	34 controls (with nTMS mapping not available)	HGG (only GBMs)	Preoperative motor mapping and nTMS-based tractography	− Influence on surgery − Perioperative complications − EOR/tumor volume − Motor function (BMRC)	To assess the impact of nTMS on the treatment strategy and clinical outcome including direct perioperative complications (at two campuses)	− In 10% of patients of the nTMS group the initial recommendation for biopsy or a “wait and see” approach was changed to resection because nTMS disproved the suspected invasion of motor structures. − Patients of the nTMS group showed a significantly higher rate of GTR. − A higher impact from nTMS was found in patients with tumors located subcortically when compared with tumors restricted to the cortex.
Hendrix et al. [63]	2016	61 adult patients	-	LGG, HGG, MET, MEN, Other	Preoperative motor mapping (110% rMT)	− Influence on surgery − EOR/tumor volume − Motor function (BMRC)	To assess the impact of nTMS on the treatment strategy and clinical outcome	− Paresis resolved or improved in 56.7% of patients one week after surgery, and 89.5% of patients with postoperative paresis improved during the follow-up interval. − Only 4.3% of patients with a metastatic lesion, but 26.3% of patients with a non-metastatic lesion experienced deterioration of motor function after surgery. − All metastatic lesions were completely resected compared with 78.9% of non-metastatic lesions.
Krieg et al. [64]	2016	120 adult patients	130 historical controls	MET	Preoperative motor mapping (110% rMT for uE and 130% for lE muscles) and nTMS-based tractography	− Influence on surgery − EOR/tumor volume − Motor function (BMRC)	To assess the impact of nTMS on the treatment strategy and clinical outcome (multi-centric with three sites)	− Patients of the nTMS group showed significantly smaller craniotomies. − Patients of the nTMS group showed a lower rate of residual tumor tissue after surgery (odds ratio: 0.3025, 95% confidence interval: 0.1356–0.6749). − Surgery-related paresis was significantly less frequent in patients of the nTMS group (nTMS group: improved: 30.8%, unchanged: 65.8%, worse: 3.4%, non-nTMS group: improved: 13.1%, unchanged: 73.8%, worse: 13.1% of patients).
Moser et al. [68]	2017	43 adult patients	-	LGG, HGG	Preoperative motor mapping (110% rMT for uE and 130% for lE muscles)	− Latency analyses − Motor function (BMRC)	To assess the impact of resection of motor-positive prerolandic nTMS points on clinical outcome	− 72% of patients showed motor-positive nTMS points in the prerolandic gyri and, thus, outside of the anatomically suspected extent of the primary motor cortex. − Out of the 13 patients who underwent resection of motor-positive nTMS points, 10 patients showed postoperative paresis (2 patients with transient and 8 patients with permanent surgery-related paresis). − Motor-positive nTMS points within the superior or middle frontal gyrus should be considered carefully and can result in motor deficits when affected during resection.
Raffa et al. [60]	2018	70 adult patients (50% also having nTMS-based fiber tracking)	35 historical controls	LGG, HGG, MET, Other	Preoperative motor mapping (120% rMT) and nTMS-based tractography	− Influence on surgery − EOR/tumor volume − Motor function (BMRC) − KPS	To assess the impact of nTMS with or without nTMS-based tractography on the treatment strategy and clinical outcome	− Patients of the nTMS and nTMS + nTMS-based tractography groups received significantly smaller craniotomies and had better postoperative motor performance and KPS scores than patients of the non-nTMS group. − Patients of the nTMS-based tractography group exhibited an improved risk-benefit analysis, a significantly increased EOR in absence of preoperative motor deficits, and significantly less motor and KPS score worsening (in case of preoperative motor deficits when compared with the nTMS group). − Risk-benefit analysis, EOR, and outcome could be improved when nTMS-based tractography is added to nTMS motor mapping.
Raffa et al. [66]	2019	79 adult patients	55 historical controls	HGG	Preoperative motor mapping (120% rMT) and nTMS-based tractography	− EOR/tumor volume − Motor function (BMRC)	To assess the impact of nTMS and nTMS-based tractography with sodium-fluorescein guidance on the treatment strategy and clinical outcome	− In patients operated on considering nTMS + FGR, the GTR rate was significantly higher compared with controls (64.5% vs. 47.2%). − Surgery-related permanent motor deficits were reduced in the nTMS + FGR group compared with controls (11.4% vs. 20%).
Raffa et al. [67]	2019	41 adult patients	41 historical controls	HGG	Preoperative motor mapping (120% rMT) and nTMS-based tractography	− Influence on surgery − EOR/tumor volume − Motor function (BMRC) − KPS	To assess the impact of nTMS and nTMS-based tractography with sodium-fluorescein guidance on the treatment strategy and clinical outcome	− Use of nTMS motor mapping and nTMS-based tractography reliably identified the spatial tumor-to-function relationship with an accuracy of 92.7%. − Patients of the nTMS group showed an increased EOR and higher rate of GTR (73.2% vs. 51.2%). − The number of cases with new surgery-related permanent motor deficits was lower in the nTMS group compared with controls (9.8% vs. 29.3%). − The number of cases with KPS worsening was lower in the nTMS group compared with controls (12.2% vs. 31.7%).
Raffa et al. [65]	2019	47 adult patients	-	MEN	Preoperative motor mapping and nTMS-based tractography	− Influence on surgery − EOR/tumor volume − Motor function (BMRC) − Arachnoidal cleavage plane	To analyze the role of nTMS motor mapping for planning resection of rolandic meningiomas and predicting arachnoidal cleavage plane.	− Use of nTMS motor mapping and nTMS-based tractography was considered useful in 89.3% of patients and changed the surgical strategy in 42.5% of patients. − A new permanent motor deficit occurred in 8.5% patients. − A higher rMT and the lack of an intraoperative arachnoidal cleavage plane were independent predictors of poor motor function outcome. − A higher rMT and perilesional edema predicted the lack of an arachnoidal cleavage plane.

Abbreviations: nTMS—navigated transcranial magnetic stimulation; LGG—low-grade glioma; HGG—high-grade glioma; MET—metastasis; MEN—meningioma; rMT—resting motor threshold; uE—upper extremity; lE—lower extremity; EOR—extent of resection; GTR—gross total resection; BMRC—British Medical Research Council; KPS—Karnofsky performance status; PFS—progression-free survival; OS—overall survival; FGR—fluorescein-guided resection; GBM—glioblastoma multiforme.

**Table 4:** Risk stratification and prediction. This table provides an overview of the studies published on risk stratification and prediction using motor mapping by nTMS (using an electric-field-navigated system) with or without additional nTMS-based tractography in patients harboring brain neoplasms.

Author	Year	Cohort	Tumor Entities	nTMS Method	Tractography Specifics	Main Objective	Main Findings
Picht et al. [69]	2012	100 adult patients	LGG, HGG, MET, MEN, Other	Preoperative motor mapping (110% rMT)	-	To provide reference values for parameters of the functional status and neurophysiological measurements	− The MEP latency was almost never different in the tumor-affected hemisphere compared with the healthy hemispheres; thus, interhemispheric differences for MEP latencies may reflect a warning sign for functional decline. − A high interhemispheric rMT ratio or a low interhemispheric MEP amplitude ratio may suggest immanent deterioration of the motor status.
Neuschmelting et al. [74]	2016	30 adult patients	HGG, MET, Other	Preoperative motor mapping (110% rMT) and nTMS-based tractography	− One ROI: motor-positive nTMS points − Deterministic tracking: FA = 100% FAT, FL = 1 mm, angular threshold of 30°	To better understand motor deficits in patients with brain tumors using structural, functional, and metabolic neuroimaging and to determine the predictive value of this approach	− Motor deficits were detected in almost all patients in whom the contrast-enhanced T1-weighted or O-(2-[18F]fluoroethyl)-L-tyrosine PET lesion area overlapped with functional tissue. − All patients who declined in motor function perioperatively showed such overlap on presurgical maps, while the absence of overlap predicted a favorable motor function outcome. − O-(2-[18F]fluoroethyl)-L-tyrosine-PET was superior to contrast-enhanced T1-weighted imaging for proposing a motor deficit before surgery. − The highest association with clinical impairment was revealed for the T2-weighted lesion area overlap with functional tissue.
Rosenstock et al. [70]	2017	113 adult patients	LGG, HGG	Preoperative motor mapping (105% rMT for uE and ~130% for lE muscles) and nTMS-based tractography	− Two ROIs: motor-positive nTMS points and ipsilateral brainstem − Deterministic tracking: FA = 75% FAT, FL = 110 mm, angular threshold of 30°	To establish risk stratification by examining whether the results of nTMS motor mapping and its neurophysiological data predict postoperative motor function outcome	− No new surgery-related permanent motor deficit was observed when the lesion-to-CST distance was >8 mm and the precentral gyrus was not infiltrated by the tumor mass. − New postoperative motor deficits were associated with a pathological excitability of the motor cortices (as indicated by an interhemispheric rMT ratio of <90% or >110%). − Motor function did not improve in any patient when the rMT was significantly higher in the tumor-affected hemisphere than in the healthy hemisphere (related to an interhemispheric rMT ratio of >110%).
Rosenstock et al. [72]	2017	30 adult patients	HGG	Preoperative motor mapping (105% rMT for uE and 130–150% for lE muscles) and nTMS-based tractography	− Two ROIs: motor-positive nTMS points and cubic ROI in the pons − Deterministic tracking: FA = 75% FAT, FL = 110 mm, angular threshold of 30°	To analyze FA and ADC within the CST in different locations and their usefulness for predicting motor function outcome	− Lower FA within the tumor-affected CST as well as higher average ADC values were significantly correlated to worsened postoperative motor function. − Segmental analyses within the CST indicated that the extent of impairment of diffusion metrics correlates with motor function deficits.
Sollmann et al. [71]	2018	86 adult patients	LGG, HGG, MET	Preoperative motor mapping (110% rMT for uE and 130% for lE muscles) and nTMS-based tractography	− Two ROIs: motor-positive nTMS points and ipsilateral brainstem − Deterministic tracking: FA = 50% FAT/75% FAT/100% FAT, FL = 110 mm, angular threshold of 30°	To explore whether nTMS-based tractography can be used for individual preoperative risk evaluation for surgery-related motor impairment	− For tractography with certain FATs, a significant difference in lesion-to-CST distances was observed between patients with HGGs who had no impairment and those who developed surgery-related transient or permanent motor function deficits. − As a cut-off value, no patient with a lesion-to-CST distance ≥12 mm showed a new surgery-related permanent paresis. − Significant negative associations were observed between the rMT and lesion-to-CST distances of patients with surgery-related transient paresis or surgery-related permanent paresis.
Seidel et al. [73]	2019	13 adult patients	LGG, HGG, MET	Postoperative motor mapping (within 14 days after surgery; “MEP loss” if not 5/10 stimulations could be elicited with 70–100% of the MSO)	-	To investigate the value of postoperative nTMS motor mapping compared with intraoperative MEP monitoring for predicting recovery of motor function	− Motor strength recovered to a score of at least 4/5 of the BMRC scale within one month after surgery if postoperative motor mapping elicited MEPs (PPV = 90.9%). − When postoperative nTMS motor mapping did not elicit MEPs, the patient was unlikely to recover in terms of motor function. − Intraoperative MEP monitoring and postoperative nTMS motor mapping were equally predictive for long-term motor recovery. − ~2/3 of patients with intraoperative MEP alterations or signal loss but positive postoperative nTMS motor mapping demonstrated motor function recovery.

Abbreviations: nTMS—navigated transcranial magnetic stimulation; LGG—low-grade glioma; HGG—high-grade glioma; MET—metastasis; MEN—meningioma; CST—corticospinal tract; ROI—region of interest; FA—fractional anisotropy; FAT—fractional anisotropy threshold; FL—fiber length; uE—upper extremity; lE—lower extremity; rMT—resting motor threshold; MEP—motor-evoked potential; PET—positron emission tomography; MSO—maximum stimulator output.

**Table 5:** Plasticity and reallocation of motor function. This table outlines the studies published on plasticity and reallocation of motor function as revealed by motor mapping using nTMS (using an electric-field-navigated system) with or without additional nTMS-based tractography in patients harboring brain neoplasms.

Author	Year	Cohort	Tumor Entities	nTMS Method	Main Objective	Main Findings
Forster et al. [75]	2012	5 adult patients (and 5 healthy volunteers)	LGG, HGG	Preoperative and follow-up motor mapping (110% rMT for uE and lE muscles; interval between mappings: 18 months on average) (two motor mappings in healthy volunteers)	To investigate cortical motor representation after resection of perirolandic WHO grade II and III gliomas	− Shift of CoGs over time was 0.7 ± 0.3 cm in the dominant and 0.8 ± 0.4 cm in the non-dominant hemisphere. − Shift of motor hotspots amounted to 0.9 ± 0.5 cm for the dominant and 0.8 ± 0.5 cm in the non-dominant hemisphere. − In one patient CoG and motor hotspot shifts significantly differed from the control group of healthy volunteers.
Takahashi et al. [76]	2013	20-year-old man	LGG	Preoperative and follow-up motor mapping (110% for uE muscles; interval between mappings: 18 months)	To confirm induced brain plasticity by nTMS motor mapping	− Primary motor representation as determined by nTMS motor mapping shifted from the precentral to the postcentral gyrus over time, which was confirmed by DES mapping. − Plastic changes in primary motor representations permitted complete tumor removal without neurological decline.
Bulubas et al. [77]	2016	100 adult patients	LGG, HGG, MET, Other	Preoperative motor mapping (110% rMT for uE and 130% for lE muscles)	To investigate whether brain lesions induce a change in motor cortex representation depending on tumor localization	− Motor areas according to nTMS motor mapping were not restricted to the precentral gyrus. − The dominant hemisphere showed a significantly greater number of longer MEP latencies than the non-dominant hemisphere. − Tumor location-dependent changes in the distribution of polysynaptic MEP latencies were observed.
Conway et al. [80]	2017	22 adult patients	LGG, HGG	Preoperative and follow-up motor mapping (110% rMT for uE and ≥130% for lE muscles; interval between mappings: 3–42 months)	To demonstrate the frequency of plastic reshaping and reveal clues to the patterns of reorganization	− Motor hotspots showed an average shift of 5.1 ± 0.9 mm on the medio-lateral axis, and a shift of 10.7 ± 1.6 mm on the antero-posterior axis. − CoGs shifted by 4.6 ± 0.8 mm on the medio-lateral axis and by 8.7 ± 1.5 mm on the antero-posterior axis. − Motor-positive nTMS points tended to shift more clearly toward the tumor if the lesion was anterior to the rolandic area than if it was located posterior to the rolandic area.
Bulubas et al. [78]	2018	100 adult patients	LGG, HGG, MET, Other	Preoperative motor mapping (110% rMT for uE and 130% for lE muscles)	To investigate the spatial distributions of motor sites to reveal tumor-induced brain plasticity in patients with brain tumors	− High MEP counts were elicited less frequently by stimulating the precentral gyrus in patients with tumors directly affecting this gyrus. − In more than 50% of these patients, the MEP counts elicited by stimulating the precentral gyrus were higher than average, indicating robust motor representations within the primary motor cortex. − Patients with parietal tumors (and specifically tumors within the postcentral gyrus) showed high MEP counts when stimulating the postcentral gyrus. − The functional reorganization seemed to be reflected by a reorganization within anatomical constraints, such as of the postcentral gyrus.
Barz et al. [79]	2018	20 adolescent to adult patients (and 12 healthy volunteers)	LGG, HGG, Other	Preoperative and follow-up motor mapping (110% rMT for uE and lE muscles; interval between mappings: 26.1 ± 24.8 months and 46.3 ± 25.4 months) (two motor mappings in healthy volunteers)	To evaluate motor cortex reorganization in patients after perirolandic glioma surgery	− Pre- and postoperatively pooled CoGs from the areas of the dominant APB muscle and non-dominant lE representation area differed significantly from those of healthy individuals. − During the follow-up period, reorganization of all muscle representation areas occurred in 3 patients, and significant shifts of uE muscle representations were detected in another 3 patients.
Sollmann et al. [81]	2018	60 adult patients	HGG	Preoperative motor mapping (110% rMT for uE and ≥130% for lE muscles) and nTMS-based tractography (and fMRI for a subsample of the cohort)	To evaluate whether brain tumor relapse has a preference to grow towards motor-eloquent brain structures	− 69.0% of patients without residual tumor, 64.3% with residual tumor away from motor areas, and 66.7% with residual tumor facing motor areas showed tumor recurrence that was directed towards motor eloquence. − Average growth towards was highest for patients with residual tumor already facing motor areas, suggesting a preference in growth patterns towards (reshaping) motor areas.

Abbreviations: nTMS—navigated transcranial magnetic stimulation; LGG—low-grade glioma; HGG—high-grade glioma; MET—metastasis; rMT—resting motor threshold; uE—upper extremity; lE—lower extremity; DES—direct electrical stimulation; fMRI—functional magnetic resonance imaging; APB—abductor pollicis brevis; CoG—center of gravity; ADM—abductor digiti minimi; MEP—motor-evoked potential; WHO—World Health Organization.

## Abbreviations

ADC	Apparent diffusion coefficient
ADM	Abductor digiti minimi
AED	Antiepileptic drug
aMT	Active motor threshold
APB	Abductor pollicis brevis
ATH	Adaptive threshold hunting
BMRC	British Medical Research Council
CoG	Center of gravity
CST	Corticospinal tract
CT	Computed tomography
DES	Direct electrical stimulation
DOF	Degrees of freedom
dMRI	Diffusion magnetic resonance imaging
EF	Electric field
EOR	Extent of resection
FA	Fractional anisotropy
FACT	Fiber assignment by continuous tracking
FAT	Fractional anisotropy threshold
FCR	Flexor carpi radialis
FDI	First dorsal interosseous
FGR	Fluorescein-guided resection
FL	Fiber length
fMRI	Functional MRI
GBM	Glioblastoma multiforme
GTR	Gross total resection
HGG	High-grade glioma
IONM	Intraoperative neuromonitoring
IR	Infrared
KPS	Karnofsky performance status
lE	Lower extremity
LGG	Low-grade glioma
MD	Mean diffusivity
MEG	Magnetoencephalography
MEN	Meningioma
MEP	Motor-evoked potential
MET	Metastasis
MNE	Minimum norm estimation
MSO	Maximum stimulator output
nTMS	Navigated transcranial magnetic stimulation
PACS	Picture archiving and communication system
PET	Positron emission tomography
PFS	Progression-free survival
pp-nTMS	Paired-pulse navigated transcranial magnetic stimulation
PTV	Planning target volume
rMT	Resting motor threshold
ROI	Region of interest
SI	Stimulation intensity
TA	Tibialis anterior
uE	Upper extremity
UT	Upper threshold
WHO	World Health Organization
